# Label-free electrochemical aptasensor for the detection of HepG2 hepatocellular carcinoma cells

**DOI:** 10.1007/s00604-024-06479-x

**Published:** 2024-06-21

**Authors:** Alexandra Pusta, Mihaela Tertis, Denisa Kezan, Diana Bogdan, Maria Suciu, Ovidiu Pană, Ionel Fizeșan, Florin Graur, Cecilia Cristea, Nadim Al-Hajjar

**Affiliations:** 1 Department of Analytical Chemistry, Iuliu Hațieganu” University of Medicine and Pharmacy, 4 Pasteur Street, Cluj-Napoca, 400349 Romania; 2 Department of Medical Devices, Iuliu Hațieganu” University of Medicine and Pharmacy, 4 Pasteur Street, Cluj-Napoca, 400349 Romania; 3 National Institute for Research and Development of Isotopic and Molecular Technologies, 67-103 Donath Street, Cluj-Napoca, 400293 Romania; 4 Electron Microscopy Centre “C. Craciun”, Biology and Geology Faculty, Babes-Bolyai University Cluj-Napoca, 5- 7 Clinicilor Str., Romania 400006 Cluj-Napoca,; 5 Department of Toxicology, Iuliu Hațieganu” University of Medicine and Pharmacy, 8 Victor Babeș, Cluj- Napoca, 400012 Romania; 6 Department of Surgery 3, Iuliu Hațieganu” University of Medicine and Pharmacy, Croitorilor, Cluj- Napoca, 19-21, 400162 Romania

**Keywords:** Electrochemical aptasensor, Functionalized screen-printed electrode, Circulating tumor cells, Hepatocellular carcinoma, HepG2 cells

## Abstract

Hepatocellular carcinoma (HCC) is the most common liver malignancy and is characterized by increasing incidence and high mortality rates. Current methods for the screening and diagnosis of HCC exhibit inherent limitations, highlighting the ever-growing need for the development of new methods for the early diagnosis of HCC. The aim of this work was to develop a novel electrochemical aptasensor for the detection of HepG2 cells, a type of circulating tumor cells that can be used as biomarkers for the early detection of HCC. A carbon screen-printed electrode was functionalized with a composite suspension containing graphene oxide, chitosan, and polyaniline nanoparticles to increase the electrode surface and provide anchoring sites for the HepG2 cell-specific aptamer. The aptamer was immobilized on the surface of the functionalized electrode using multipulse amperometry, an innovative technique that significantly reduces the time required for aptamer immobilization. The innovative platform was successfully employed for the first time for the amplification-free detection of HepG2 cells in a linear range from 10 to 200,000 cells/mL, with a limit of detection of 10 cells/mL. The platform demonstrated high selectivity and stability and was successfully used for the detection of HepG2 cells in spiked human serum samples with excellent recoveries.

**Graphical abstract:**

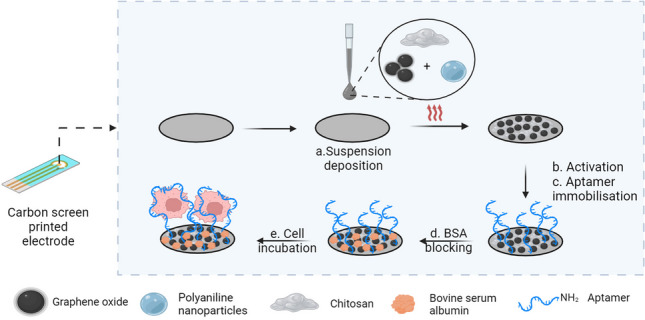

**Supplementary information:**

The online version contains supplementary material available at 10.1007/s00604-024-06479-x.

## Introduction

Liver cancer is a malignancy with increasing incidence and high mortality rate, being the third cause of cancer-related mortality globally [1]. The most common type of liver cancer is hepatocellular carcinoma (HCC), which accounts for up to 85% of all liver cancer cases [2]. The high mortality rate of HCC is in part due to the lack of specific symptoms in the early stages of the disease, which leads to late diagnosis and poor clinical outcomes [3]. To help the early diagnosis of HCC and improve the patient survival rate, new screening methods are needed.

Circulating tumor cells (CTCs) are cancer cells that shed from the primary tumor into the blood stream, migrating to secondary organs and generating metastases [4]. CTCs are present in peripheral blood in low concentrations, ranging from 1 to 3000 cells/mL [5]. HepG2 cells are CTCs that can be present in the blood of patients suffering from HCC. The detection of CTCs by minimally invasive methods could lead to better cancer diagnosis and management. Currently, different methods can be used for the analysis of CTCs, such as immuno-affinity assays [6–8], size-dependent separation [9], fluorescence imaging [10], and electrochemical sensors [5, 11–14]. The latter have attracted attention in the last years, due to their rapidity, simplicity, need for small sample volumes, and portability.

To increase the selectivity of electrochemical sensors, biological or biomimetic elements such as aptamers can be used to functionalize the electrode surface [15]. Aptamers have become widely used in the field of electrochemistry, due to their advantages such as higher stability compared to antibodies, long shelf life, and ease of synthesis and functionalization [16]. The integration of aptamers in electrochemical sensors generates aptasensors, which exhibit high specificity for the target analyte. Aptamer immobilization on the electrode surface is generally done by incubating the aptamer solution on the electrode surface and allowing them to interact for a period of time. This procedure is sometimes lengthy and extends the duration of the aptasensor fabrication protocol. An alternative, faster immobilization method consists in the use of multipulse amperometry to facilitate aptamer functionalization. This method has been successfully applied in our group for the functionalization of gold electrodes/gold structures with thiolated aptamers [17]. Given the promising results obtained in previous studies, in this work, we tested, for the first time, the immobilization of an amino-terminated aptamer via multipulse amperometry-assisted incubation.

Several aptasensors have been developed for the detection of HepG2 CTCs. Sun et al. developed a sandwich electrochemical approach for the detection of HepG2 cells. Gold electrodes modified with aptamer TLS11a were used for the capture of HepG2 cells, while gold nanoparticles modified with hemin, aptamer, and horseradish peroxidase acted as a nanoprobe for signal amplification [11]. The same group reported a similar sandwich approach, using indium tin oxide electrodes and gold/palladium-functionalized ZnO nanorods as nanoprobes [12]. Another approach consisted in the integration of TLS11a aptamer into a DNA tetrahedron structure which was then immobilized onto gold electrodes. The detection was carried out using a sandwich approach based on multibranched hybridization chain reaction amplification [5] or by the use of nanoprobes consisting of Pt-Pd nanocages that lead to the formation of dendritic structures and to enhanced signal [13]. Similar sandwich techniques were also developed for the electrochemical detection of exosomes derived from HepG2 cells [14].

Nanomaterials are used to modify electrode surfaces to increase sensitivity and to act as anchoring sites for biological or biomimetic elements. Among nanomaterials, carbon-based materials are frequently used in electrochemistry due to their characteristics. Graphene oxide (GO) is a 2D single atom layer of carbon which contains numerous oxygen containing functional groups [18, 19]. Despite its poor electrical conductivity, GO can be successfully used in the development of electrochemical aptasensors because the carboxyl groups in its structure provide attachment sites for aptamers, allowing aptamer functionalization and immobilization [18–20]. Moreover, GO is highly hydrophilic which makes it compatible with aqueous solutions used in electrochemistry [21, 22]. Polymeric solutions can be used to form nanomaterial suspension that can be drop-cast onto the electrode surface. Chitosan (Chi) is a polymer derived from chitin, which exhibits high biocompatibility and film-forming properties. At acidic pH, the amino groups in the structure of chitosan are protonated, making chitosan a cationic compound with electrical conductivity, which makes it promising for electrochemical applications [23]. Polyaniline stands as one of the most versatile conducting polymers, with several merits such as simple preparatory process, robust environmental stability, heightened conductivity, and its compatibility for composite formation with various materials [24–26]. Nanostructured polyaniline has garnered significant interest owing to its key role in the development of electronic devices and sensors as well as for biomedical applications. Among the nanostructured polyaniline variants, nanoparticles (NPs) hold particular prominence. These NPs can be synthesized through diverse techniques, including soapless emulsion polymerization, sol-gel and ultrasound-assisted sol-gel approaches, precipitation or coprecipitation with subsequent heat treatment, or the use of templates to guide NP formation [24, 25].

In this work, a novel composite of GO, Chi, and polyaniline NPs was applied in the design of an aptasensor for the detection of HepG2 cells. Each component presented a specific contribution: polyaniline NPs augmented the polymeric film, yielding additional space for GO to be exposed, thus allowing improved aptamer immobilization and leading to heightened sensitivity. Polyaniline NPs were chosen due to their recognized electrical conductivity, aiming to enhance electron transfer at the surface and counterbalance the less conductive properties of GO, also present in the composite film. The carboxyl groups in the structure of GO were activated to allow the covalent bonding of the amino-terminated TLS11a aptamer on the surface of GO [27].

An innovative strategy based on multipulse amperometry was applied for aptamer immobilization via amide bond. This procedure greatly reduces the length of the aptasensor development process. Lastly, the aptasensor was incubated with HepG2 cells, selected as biomarkers for the fast screening of HCC. Each surface modification step was analyzed by cyclic voltammetry (CV) and electrochemical impedance spectroscopy (EIS). HepG2 cells were detected in this study using EIS as a label-free method, following a less than 100-min protocol, starting with electrode activation, followed by aptamer immobilization, blocking, cell incubation, and detection. The detection method was applied on suspensions containing HepG2 cells as well as to spiked human serum samples with good recoveries. The reported method is fast and simple due to the innovative use of multipulse amperometry-assisted aptamer deposition and presents high selectivity for the detection of HepG2 cells. Compared with other electrochemical aptasensors for HepG2 cells reported in the literature, this approach does not require signal amplification strategies while maintaining good analytical performance.

## Materials and methods

All the details about the reagents, materials, and instruments used in work are presented in the supplementary material.

### Methods

#### Aptamer solution preparation

See details in the supplementary material.

#### Polyaniline NPs synthesis

Polyaniline NPs were synthesized in a bulk solution using a method previously described in the literature, involving the oxidative polymerization of aniline and ammonium peroxodisulfate as an oxidant in aqueous medium, without the use of any acid [24]. In brief, 7 mmols of aniline were combined with 20 mL of ultrapure water and subjected to magnetic bar stirring for 10 min. The mixture was then maintained at 5 °C for 30 min, followed by additional stirring. A solution containing 7 mmol of ammonium peroxodisulfate prepared in 80 mL of ultrapure water was added in one portion to the aniline solution. The resulting mixture was stirred for an additional hour to ensure thorough mixing. This solution was then kept at 5 °C for 24 h. The resultant polymer was collected on filter paper, washed with ultrapure water, and dried under vacuum at 50 °C for a day to yield polyaniline emeraldine salt. The polymer was rinsed with an excess of 0.1-M NH_4_OH, collected, and dried as previously described to obtain the deprotonated form, emeraldine base. A suspension of 0.3 mg/mL of polyaniline NPs was prepared by homogenizing the appropriate quantity of solid in ethanol.

#### GO/Chi/NP suspension preparation

The necessary quantity of solid Chi was weighed and dissolved in HCl 0.5 M to obtain a 2% Chi solution. The solution was sonicated for 1 h and was then stirred using a magnetic stirrer at 500 rpm for 2 h. The obtained Chi solution was mixed with the commercial 4 mg/mL GO suspension in a ratio of 1:3 using a magnetic stirrer. The GO suspension was sonicated for 30 min prior to mixing with the Chi solution. Finally, the GO/Chi suspension was mixed with the NP suspension prepared in ethanol, in a ratio of 2:1. The obtained GO/Chi/NP suspension was stored at 4 °C and was used to modify the electrodes.

#### Aptasensor fabrication

The C-SPE was first functionalized with the obtained GO/Chi/NPs suspension. The suspension was applied in two layers of 2.5 µL each, and each layer was allowed to air dry at room temperature before the application of the next one. After the application of the last layer, the electrodes were dried in an oven at 50 °C for 30 min. The obtained C-SPE/GO/Chi/NPs were then activated using a mixture of 13.33 mM N-hydroxysuccinimide (NHS) and 6.66-mM 1-ethyl-3-(3-dimethylaminopropyl)carbodiimide (EDC) dissolved in DNAse/RNAse-free water. A total of 5 µL of the NHS/EDC mixture were applied onto the working electrode and incubated for 30 min in a humid environment. After activation, the excess NHS/EDC mixture was washed three times with 200-µL TRIS buffer to remove the unreacted activation agents. Next, 30 µL of 2-µM aptamer solution was applied to the electrode and immobilized via multipulse amperometry to obtain C-SPE/GO/Chi/NPs/Apt. Multipulse amperometry was performed in the following conditions: 9000 series alternating the application of 0.5 V for 10 ms and −0.1 V for 10 ms for 180 s. After this step, the electrodes were washed three times with 200-µL TRIS to remove the unbound aptamers. To block the remaining unoccupied sites and prevent unspecific adsorption, the electrode surface was blocked with a 0.5% BSA solution prepared in TRIS buffer. The electrodes were left in contact with the BSA solution for 30 min in a humid atmosphere to obtain C-SPE/GO/Chi/NPs/Apt/BSA and were then washed three times with 200-µL TRIS. The 30-min duration of the blocking step was considered reasonable; thus, multipulse amperometry-assisted blocking was not applied to avoid aptamer detachment. Finally, 5 µL of cell suspensions prepared in PBS pH 7.4 of different concentrations was incubated onto the modified electrodes for 30 min, followed by washing and electrochemical detection. EIS and CV analyses were performed after each step in the aptasensor fabrication. EIS was carried out in a 2-mM [Fe(CN)_6_]^4−/3−^ solution prepared in 0.1-M KCl, using the following parameters: 61 frequencies from 0.1 to 100,000 Hz and amplitude 0.01 V at open circuit potential (OCP). The step-by-step functionalization of the electrode surface was also investigated by CV in 2-mM [Fe(CN)_6_]^4−/3−^ by varying the potential between − 0.5 V and 1 V for two cycles with a scan rate of 100 mV/s.

Different parameters, such as the number of suspension layers applied (1, 2, 3) on the electrode surface, the activation times (15, 30, and 60 min), the aptamer immobilization protocol (simple incubation and multipulse-assisted immobilization), the concentration of aptamer used for incubation (0.5, 1, and 2 µM), and the duration of the blocking procedure (15, 30, and 60 min), were tested, and the optimized method was described above.

#### Cell culture and detection of cancerous cells

Cancerous hepatic HepG2 cells and human lung adenocarcinoma A549 cells were cultured in EMEM with low glucose and in DMEM with high glucose, respectively. The normal fibroblast BJ cells were cultured in DMEM with low glucose. For all cell types, the complete media were supplemented with 10% fetal bovine serum (FBS). Cells were kept in a humidified atmosphere with 5% CO_2_ supplementation at 37 °C, and the media was changed every other day. At an approximate confluency of 70–80%, all cell types were trypsinized, resuspended in culture medium, and centrifuged at 300 g for 10 min. Consequently, the media were removed, and the cell pellets were resuspended in PBS. Cells were counted using Burker-Turk counting chambers and trypan blue exclusion to eliminate dead cells from the count. The cell suspensions in PBS were further diluted with PBS to achieve a concentration of 200,000 cells/mL that was used for testing the aptasensor performances.

The detection of HepG2 cells was carried out after the incubation of HepG2 cell suspensions prepared in PBS pH 7.4 with the aptasensing platform for 30 min. After incubation, the electrodes were washed, and EIS was performed in a 2-mM [Fe(CN)_6_]^4−/3−^ solution prepared in 0.1-M KCl, using the following parameters: 61 frequencies from 0.1 to 100,000 Hz and amplitude 0.01 V at open circuit potential (OCP). The R_ct_ was recorded before and after cell incubation, and the difference ΔR_ct_ was calculated as [R_ct_ after cell immobilization — R_ct_ before cell immobilization]. The obtained ΔR_ct_ values were plotted against the cell concentration, and the calibration curve was obtained. All tests were performed in triplicate.

#### SEM, AFM, and XPS characterization

See details in the supplementary material.

#### Selectivity, stability, and real sample analysis

The selectivity of the aptasensor towards HepG2 cells was tested by incubating the aptasensor with fibroblasts and A549 cells (200,000 cells/mL in PBS) and performing electrochemical tests before and after incubation. Moreover, to demonstrate the selectivity of the aptamer for HepG2 cells, another aptamer sequence 5′NH_2_-TGC-GTG-TGT-AGT-GTG-TCT-GTT-GTT-TGT-ATT-GTT-GTC-TAT-CCT-CTT-AGG-GAT-TTG-GGC-GG-3′ (Eurogentec, Seraing, Belgium) was used to modify the electrodes, using the same protocol described in Sect. 2.1 in the supplementary material. The aptasensor was then incubated with HepG2 cells, and electrochemical tests were performed.

The stability of the aptasensor was tested by preparing multiple aptasensors on the same day and testing them via EIS in 2-mM [Fe(CN)_6_]^4−/3−^ on consecutive days. The aptasensors were kept at 4 °C until use.

Undiluted commercial human serum was spiked with known concentrations of HepG2 cells, and EIS and CV analyses were performed. All tests were done in triplicate, and the recoveries were calculated for all tested concentrations. The ΔR_ct_ values were calculated and plotted against the cell concentration, and a calibration curve in serum was obtained.

## Results and discussions

### Aptasensor fabrication

The schematic representation of the step-by-step aptasensor fabrication protocol is presented in the Graphical abstract. The bare C-SPE was functionalized by drop-casting with a suspension containing GO, NP, and Chi. The carboxyl groups in the structure of GO are further used as anchoring sites for aptamer immobilization. To achieve this, the carboxyl groups were activated using NHS/EDC to allow the formation of amide bonds between the activated carboxyl groups and the amino group at the 5′ end of the aptamer sequence. EDC was used as a cross-linker that forms an intermediary reaction product that can then react with the primary amino group. NHS is used to increase the stability of the intermediary product, by converting it to a second, more stable intermediary product before the amination reaction takes place [28]. Aptamer immobilization was performed using an optimized multipulse amperometry-assisted method that seems to facilitate the formation of the amidic bond. After aptamer immobilization, the remaining unoccupied spaces on the surface of the aptasensor were blocked using BSA. Lastly, the aptasensor was incubated with HepG2 cells which interacted with the aptamers immobilized onto the electrode surface. This interaction leads to conformational changes in the structure of the aptamer, leading to changes in the Nyquist spectra obtained by EIS. The increase in R_ct_ with the concentration of cells was investigated using increasing concentrations of HepG2 cells.

### Platform optimization

For the development of the aptasensor, the following parameters were optimized: (1) the number of suspension layers deposited on the electrode, (2) the activation time, (3) the aptamer immobilization protocol, (4) the aptamer concentration, and (5) the blocking duration. CV and/or EIS were performed for each parameter to determine the optimal conditions. The results are presented in Table 1 with the parameters chosen as optimal written in bold.

**Table 1:** Results obtained in EIS and CV for each optimization step

Fabrication step	Optimized parameter		*R*_ct_ (Ω)	I_ox_ (µA)
Suspension deposition	Number of suspension layers (2.5 µL/layer)	Bare	1409	91
		1	147	128
		**2**	**20**	**230**
Activation	Activation time	15 min	26	223
		**30 min**	**43**	**215**
		60 min	48	213
Apt immobilization	Immobilization procedure	Simple incubation (1 h, aptamer concentration 2 µM)	135	209
		**Multipulse amperometry** **(180 s, 2 µM)**	**215**	**191**
	Apt concentration (multipulse amperometry, 180 s)	0.5 µM	73	211
		1 µM	137	204
		**2 µM**	**215**	**191**
BSA blocking	Blocking time (BSA 0.5%)	15 min	516	168
		**30 min**	**1098**	**152**
		60 min	1103	149

The GO/Chi/NPs suspension was deposited on the electrode surface in layers of 2.5 µL each. It can be observed from both EIS and CV data that a sharp increase in surface conductivity was noticed after the application of one layer, followed by an even more marked increase in conductivity after the application of two layers. Further functionalization of the surface with additional layers led to the detachment of the polymeric film after electrochemical testing, so two layers were chosen as the optimal number which ensures maximum conductivity and stability. Activation with NHS/EDC for 15 min led to minor changes in EIS and CV; however, after 30 min of activation, the measured R_ct_ doubled compared to the previous fabrication step. Doubling the activation time to 60 min did not result in significant changes neither in R_ct_ nor in I_ox_, so 30 min were chosen as the optimal activation time. Aptamer immobilization was optimized with respect to the procedure used and aptamer concentration. Multipulse amperometry-assisted immobilization from a 2-µM aptamer solution provided a 60% increase in R_ct_ compared to a 1-h simple incubation protocol. Longer incubation times were not tested, since the multipulse-assisted amperometry process provided satisfactory immobilization in a very short time (3 min). The differences obtained for the three different aptamer concentrations were not very noticeable in CV; however, EIS is a more sensitive technique, and, in this case, a proportional increase in R_ct_ was noticed with the increase of the aptamer concentration. EIS data was used for the optimization of this step, and 2 µM was chosen as the optimal concentration since it provided the highest increase in R_ct_. Blocking of the electrode surface with BSA 0.5% for 30 min doubled the measured R_ct_ compared to blocking for only 15 min, while a further doubling of the activation time to 60 min did not result in significant differences; thus, the optimal blocking time was 30 min.

### Electrochemical characterization

Each step in the fabrication of the aptasensor was characterized by CV and EIS in 2-mM [Fe(CN)_6_]^4−/3−^ prepared in 0.1-M KCl.

**Fig. 1:**
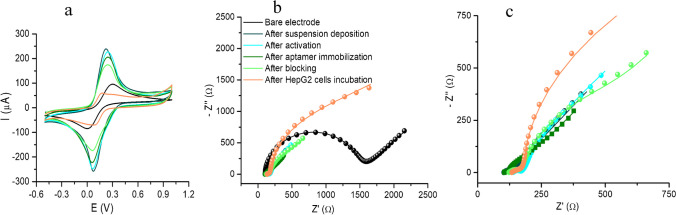
Step-by-step electrochemical characterization of the electrode surface performed in a 2-mM [Fe(CN)_6_]^4−/3−^ solution by CV (**a**) and EIS (**b**) and (**c**) on bare C-SPE (black), after GO/Chi/NP suspension deposition (dark blue), after NHS/EDC activation (cyan), after aptamer immobilization (dark green), after BSA blocking (light green), and after HepG2 cell immobilization (orange). The Nyquist representation of the EIS data was depicted with points, while the fit and simulation data (model) was depicted as solid line

In CV, the characteristic oxidation and reduction peaks of the redox probe were obtained in the case of the bare C-SPE, with a peak-to-peak separation of 0.283 V (Fig. 1a, black). After suspension deposition, an increase in the peak intensities as well as a reduction in peak-to-peak separation (0.146 V) could be observed (Fig. 1a, dark blue). This behavior can be attributed to the electroconductive nature of chitosan and the polyaniline NPs [24–26], as well as to the increase of the electroactive surface after suspension deposition. Next, surface activation with NHS/EDC led to a slight decrease of the current intensity and a slight increase in peak-to-peak separation (0.171 V) (Fig. 1a, cyan). All subsequent steps: aptamer immobilization, BSA blocking, and HepG2 cell incubation (Fig. 1a dark green, light green and orange) led to progressively lower current intensities but lower peak-to-peak separation (0.177 V, 0.173 V, 0.093 V).

The electrochemical behavior determined by CV was also confirmed by EIS.

Figure 1b displays the Nyquist diagrams illustrating the impedance data. In the case of the bare C-SPE, an R_ct_ of 1409 Ω was registered in 2-mM [Fe(CN)_6_]^4−/3−^(Fig. 1b, black). After suspension deposition, there was a marked decrease in the R_ct_ reaching a value of 20 Ω (Fig. 1b, dark blue). The electroconductive components of the suspension greatly improve the conductivity of the electrode surface [21, 24, 26]. Next, the activation of carboxyl groups using NHS/EDC led to a slight increase in R_ct_ to 43 Ω (Fig. 1b, c, cyan). After immobilization of the aptamer via multipulse amperometry, the R_ct_ was significantly increased to 215 Ω, demonstrating the successful binding of the aptamer on the modified electrode surface (Fig. 1b, c, dark green). The negative charges in the aptamer structure repel the redox probe, leading to hindered electron transfer and a higher R_ct_. BSA incubation increased the R_ct_ to 1098 Ω, due to the size of the BSA molecule which sterically hinders the access of the redox probe to the electrode surface (Fig. 1b, light green) [29]. HepG2 cell incubation further increased the R_ct_ to the highest value, 2765 Ω (Fig. 1b, c, orange). This confirms the ability of the aptasensor to bind HepG2 cells. It was noticed that the increase of R_ct_ after HepG2 cell incubation is proportional to the cell concentration; therefore, an impedimetric method was used for their detection. The detection of HepG2 was achieved by calculating the difference between the R_ct_ after and before HepG2 (ΔR_ct_) cell incubation and plotting it versus the cell concentration.

The EIS experimental data were fitted using the Randles equivalent circuit to determine the simulated values of kinetic parameters (see all the corresponding components of the equivalent circuits in Table S1). The components of the circuit, represented schematically as [R_Sol_(CPE[R_ct_W])], include the following: the solution resistance (R_Sol_), which encompasses equipment and electrical circuit element resistance, the charge-transfer resistance (R_ct_), the double-layer capacitance expressed here through the constant phase element (CPE), and the Warburg impedance (W). The replacement of the double-layer capacitance with CPE may be due to the fact that in practical situations, EIS representations often deviate from the theoretically expected patterns and exhibit distinct distortions, such as depressed semicircles that can be described more accurately by CPE [30].

After the electrode’s functionalization with the composite film containing GO, Chi, and NP (dark blue), activation of the carboxyl groups via NHS/EDC chemistry (cyan), immobilization of the aptamer (dark green), blocking step with BSA (light green), and after HepG2 cell immobilization (orange), the Nyquist diagrams of EIS exhibit three distinct regions: a semicircle in the high-frequency range, another semicircle in the intermediate part of the frequency spectrum, and a line forming an angle of approximately 45° with the Ox axis (Z′-axis, expressed in kΩ) at lower frequencies. The third component in the list pertains to diffusion, involving the movement of redox species through the electrolyte towards the electrode surface and vice versa, emanating from the electrode into the bulk [31]. The initial semicircle, evident at higher frequencies, reflects the resistance of the interface layer, denoted here as R_ct_. It is well known that the smaller semicircle indicates lower impedance, thereby facilitating charge transfer, while the larger semicircle indicates higher impedance, impeding charge transfer. The second semicircle of the Nyquist diagram is possibly due to the formation of an electrical double layer involving ion diffusion. This is associated with constrained ion movement, uneven pore diameter of the electrode, and surface roughness. Such a phenomenon is typically observed in electrochemical processes with more than one rate-determining step [32]. This reveals the non-uniformity and high porosity of the C-SPE after different functionalizations performed during the aptasensor elaboration protocol. The success of the activation of the carboxyl groups on GO as well as the immobilization of the aptamer can also be demonstrated by the changes observed in the kinetics at the electrode interface, highlighted by the modification of the equivalent circuit and the introduction of a series of R and C elements connected in parallel. For the blocking step with BSA and the final step of the experimental protocol, the immobilization of the analyte onto the electrode, the equivalent circuit used for modeling the experimental EIS data was modified again. This modification introduced another series of R and C elements connected in parallel that could be attributed to alterations in the electron transfer mechanism at the electrode surface, as well as the successful immobilization of BSA molecules and HepG2 cells, respectively.

Further details regarding the proposed equivalent circuits for each stage of the experimental protocol, including the constituent elements and the parameters that describe them, are provided in the supplementary files in Table S1 and Table S2. It can also be observed in Table S2 that the *χ*^2^ values are relatively small, indicating that the data fits well the selected circuits.

### Surface characterization

#### SEM characterization of the aptasensor

**Fig. 2:**
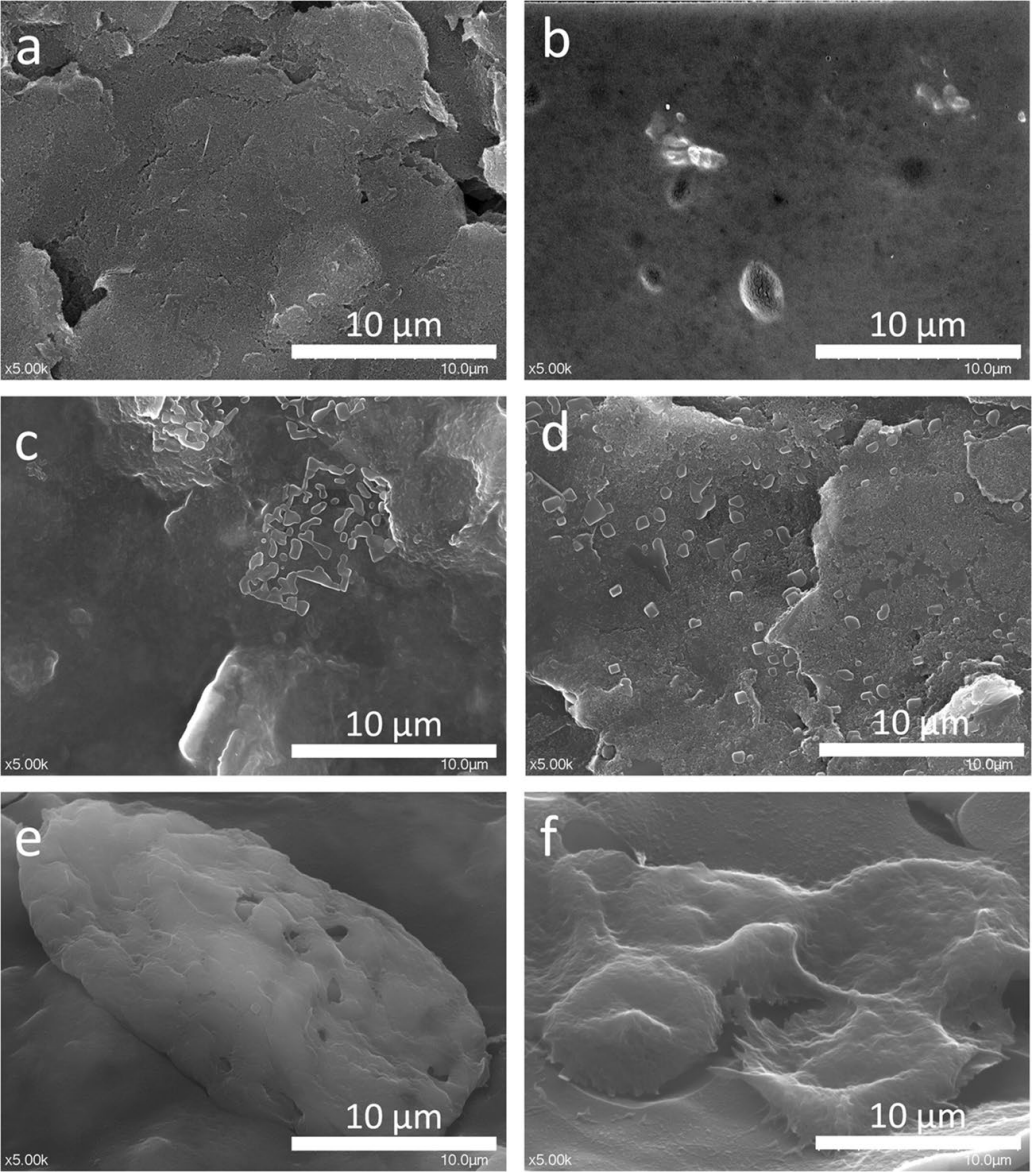
SEM images of the unmodified C-SPE (**a**), C-SPE/GO/Chi/NPs (**b**), C-SPE/GO/Chi/NP/Apt (**c**, **d**), and C-SPE/GO/Chi/NP/Apt with immobilized HepG2 cells (**e**, **f**)

SEM analyses were performed to characterize the surface of the electrode after each modification step. Figure 2 shows the unmodified C-SPE with the typical flaky appearance (Fig. 2a).

Deposition of the GO/Chi/NP suspension changed the surface topography to a thin and smooth layer of polymeric appearance (electron semi-transparent) (Fig. 2b). When the aptamer was also added to the modified electrode, the surface was drizzled with small clusters of crystallites (Fig. 2c & d), and HepG2 cells were able to attach and expand on these surfaces (Fig. 2e & f). After immobilization on the electrode surface, a slight change can be seen in the shape of the cells, which stretches in the XOY plane, following the profile of the electrode. This behavior was also observed for other types of cells immobilized via the interaction with a specific aptamer [20]. The measured size of the HepG2 cells present at the electrode surface corresponds to the 10–20-μm size described in the literature [33].

#### AFM characterization of the aptasensor

**Fig. 3:**
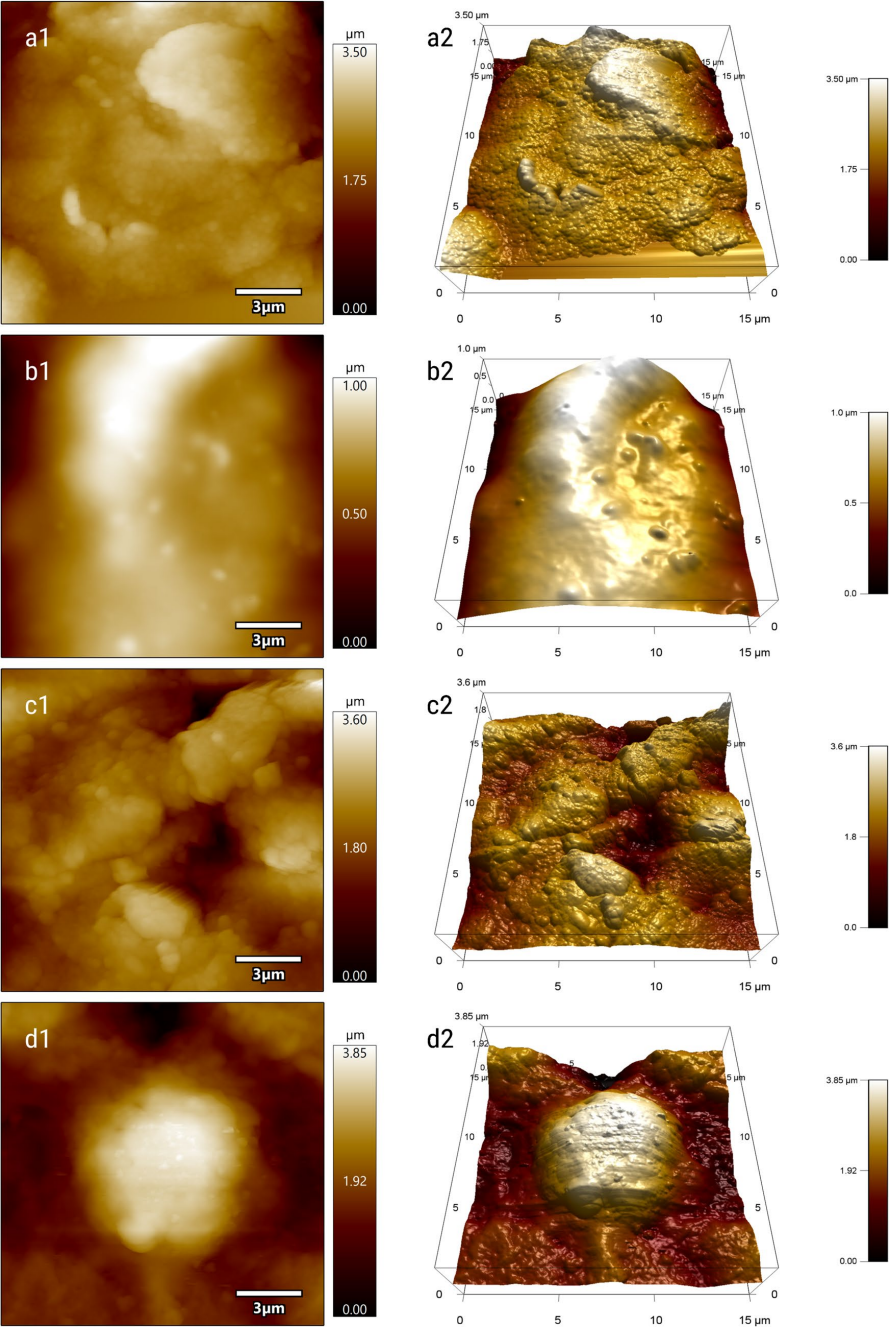
AFM characterization. 2D (left) and 3D (right) AFM topographic images of unmodified C_SPE (**a1 and a2**), C-SPE/GO/Chi/NPs (**b1 and b2**), C-SPE/GO/Chi/NPs/Apt (**c1** and **c2**), and C-SPE/GO/Chi/NPs/Apt with immobilized HepG2 cells (**d1** and **d2**). Scan size 15 μm

The electrode surface was analyzed in terms of morphology and topography using AFM after each consecutive modification step. AFM images show distinctive characteristics of the bare (Fig. 3a1 and a2) and, respectively, modified electrode, after each step.

A smoother surface of the electrode is evident for SPE_GO/Chi/NP as compared to the bare electrode, due to the polymeric chitosan (Fig. 3b1 and b2). When the aptamer is added, the roughness of the electrode surface increases, indicating a successful functionalization (Fig. 3c1 and c2). The immobilization of the HepG2 cells takes place with a slightly deformation of the cell shape, which tends to adapt to the surface morphology (Fig. 3d1 and d2). This behavior was also noticed in other previously published studies [20]. The cells are rare at the electrode surface and are positioned predominantly in valleys. AFM results also confirmed the diameter of the cells as it can be observed in Figure S1.

#### XPS characterization of the aptasensor

**Fig. 4:**
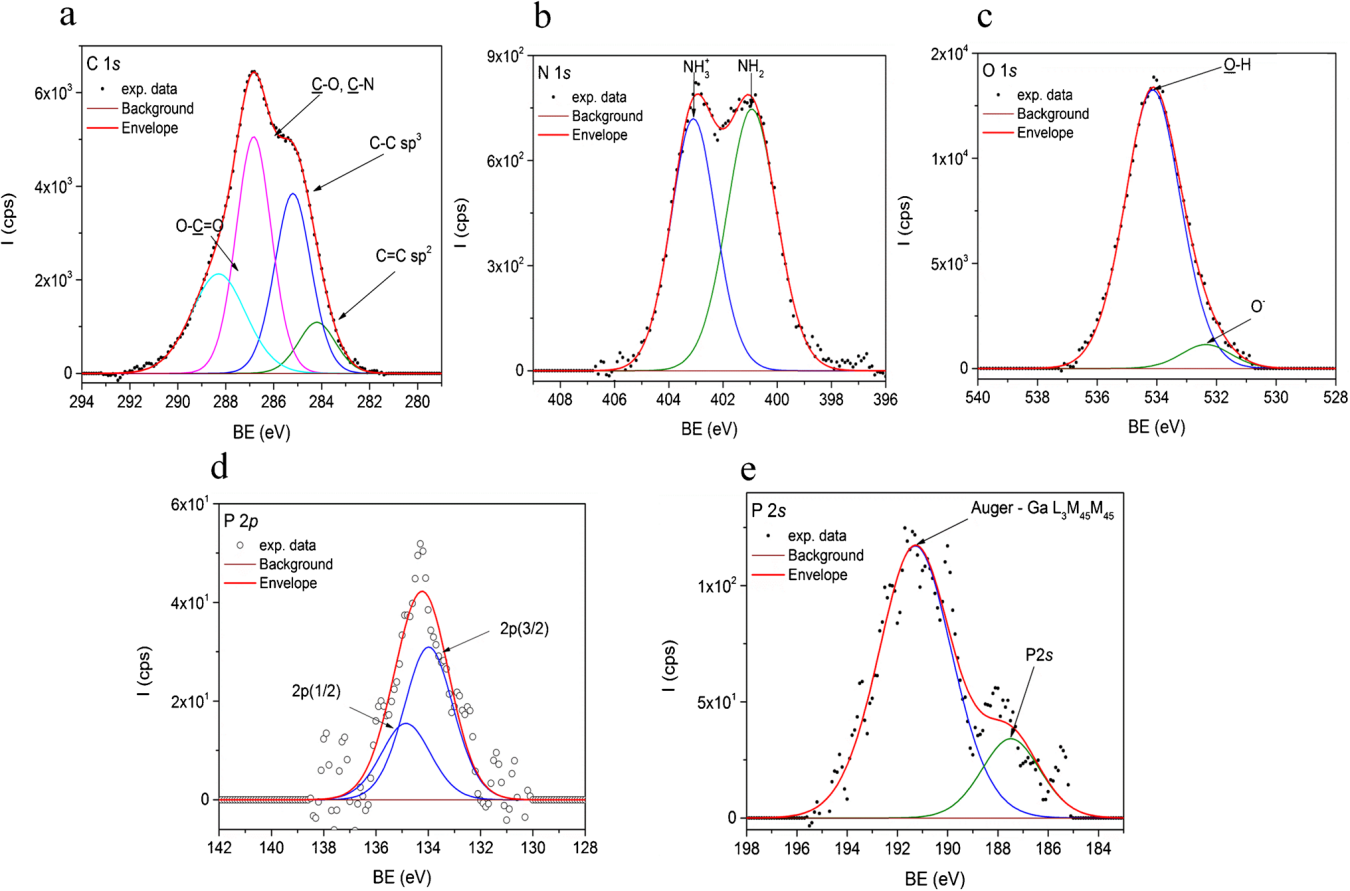
XPS data obtained for the C-SPE/GO/Chi/NP platform: C 1s core-level spectrum of graphite/chitosan sample electrode (**a**), N 1s core level lines of the amine/amide groups at and of the specific protonated amine, NH_3_^+^ determined in case of graphite/chitosan electrode (**b**); O 1s core-level lines obtained for graphite/chitosan electrode (**c**); C-SPE/GO/Chi/NP/Apt platform: XPS spectrum of the 2p deconvoluted core-level line of phosphorus from the aptamer containing electrode (**d**); XPS spectrum of the 2s core-level line of phosphorus due to the aptamer presence (**e**). The normalized area corresponds to the P 2p(3/2) normalized area from Fig. 4d

The XPS characterization of the aptasensor platform was performed at all functionalization steps. Figure 4a shows the C 1s core-level spectrum of graphite/chitosan sample electrode. Here the integral intensity of the carboxyl line is significantly increased indicating the formation of new covalent functionalization between the COOH on the GO sheets and the N groups of chitosan molecule in the composition of the suspension.

Thus, when chitosan is deposited onto the graphite electrode, its specific N 1s core-level peaks are observed (Fig. 4b) with two positions of N 1s core level: the amine and amide groups at a BE of ~ 400.9 eV and the protonated amine, NH_3_^+^, at a BE of 403.0 eV. Very interesting, the protonation of the amine can be associated with the appearance of an O 1s peak at lower BE associated with the formation of carboxylate anion (Fig. 4c). The ratio between NH_3_^+^ and the O^−^ normalized intensities is 0.98. It appears that chitosan film is attached to the graphite substrate through ionic bonds. Generally, in acidic solution, amine groups of chitosan are protonated to NH^3+^, and the fraction of free amine groups is a key parameter to understand this material mechanisms of binding onto various surfaces [34, 35].

The presence of the aptamer on the modified electrode can be qualitatively highlighted by the existence, in the XPS spectrum, of the 2p and 2s core-level lines of phosphorus, as well as by the correlation between their areas, A_2p_/A_2s_. The results are shown in Fig. 4d and e. The determined ratio between areas of P 2p (3/2) and P 2s is ~ 1, thus showing the consistency of the quantification. The large feature observed in Fig. 4e at higher BE is due to gallium Auger emission L_3_M_45_M_45_ with a kinetic energy of ~ 1062 eV and possibly comes from the contact electrodes alloy (unknown composition); thus, the quantification of the C 1s, N 1s, and O 1s core levels, even if it can be performed, contains a high degree of uncertainty, being irrelevant.

### Analytical performance

**Fig. 5:**
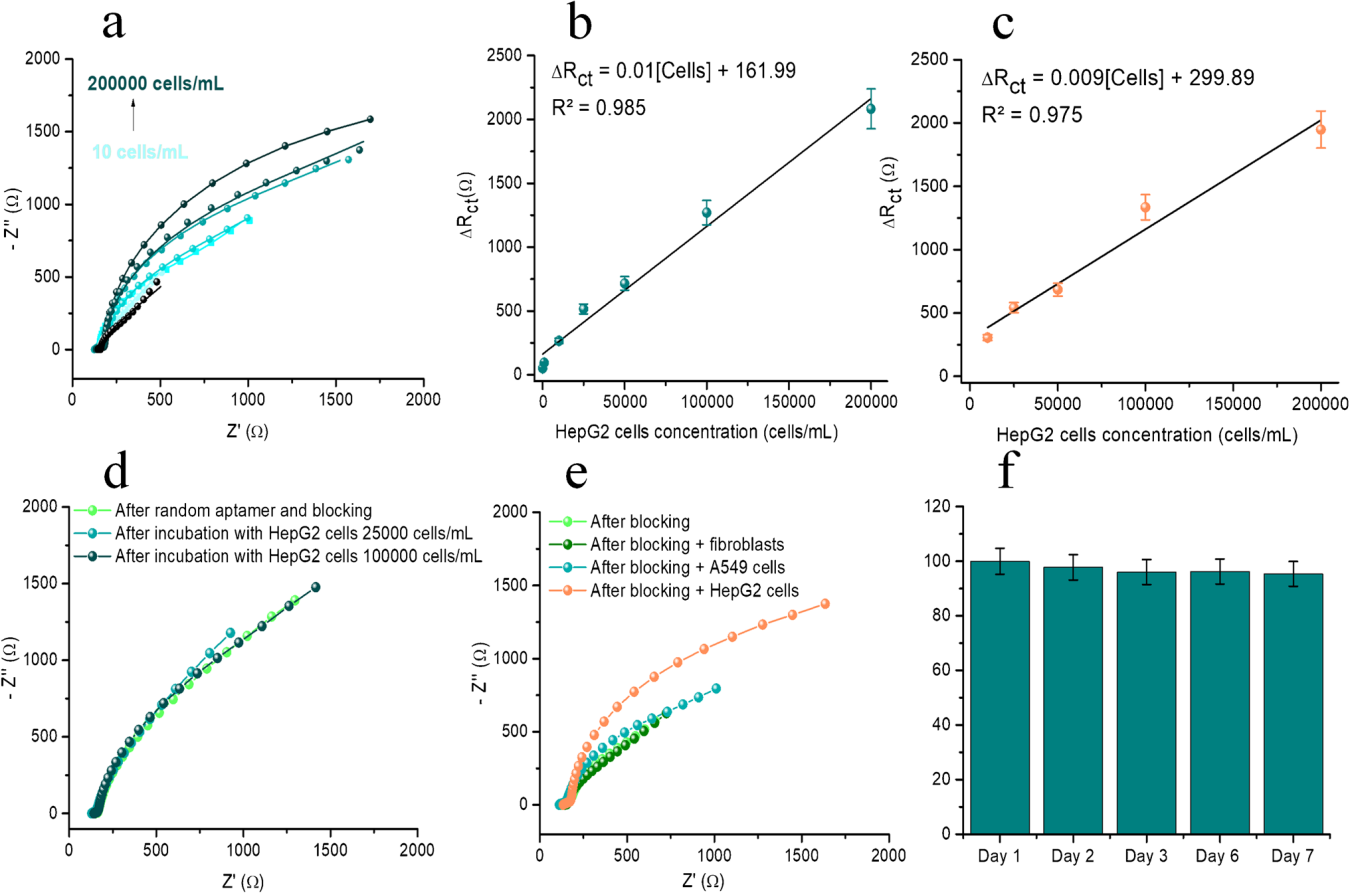
Nyquist plots obtained in 2-mM [Fe(CN)_6_]^4−/3−^ for increasing concentrations of cells: 10, 1000, 10,000, 25,000, 50,000, 100,000, and 200,000 cells/mL (light to dark blue) and blank PBS pH 7.4 (black) (**a**). Calibration curve obtained for the domain 10–200,000 HepG2 cells/mL in PBS. Error bars represent the standard deviation obtained for three different tests (**b**). *Real sample analysis* calibration curve obtained for the domain 10,000–200,000 HepG2 cells/mL in undiluted serum. Error bars represent the standard deviation obtained for three different tests (**c**). *Selectivity* Nyquist plots obtained in 2-mM [Fe(CN)_6_]^4−/3−^ after immobilization of a random DNA sequence on the electrode surface and BSA 0.5% blocking (light green) followed by incubation with HepG2 cell at 25,000 cells/mL (light blue) and 100,000 cells/mL (dark blue) (**d**). Nyquist plots obtained in 2-mM [Fe(CN)_6_]^4−/3−^ after immobilization of TLS11a aptamer on the electrode surface and BSA 0.5% blocking (light green) followed by incubation with fibroblasts at 2,000,000 fibroblasts/mL (dark green) and HepG2 cells at 2,000,000 HepG2 cells/mL (orange) (**e**). *Stability* ΔR_ct_ obtained on the aptasensing platform on days 1, 2, 3, 6, and 7 (**f**)

The analytical performance of the aptasensor was evaluated in a 2-mM [Fe(CN)_6_]^4−/3−^ solution using EIS. Seven different cell suspensions of different concentrations in the range 10–200,000 cells/mL (prepared in PBS pH 7.4) were incubated with the aptasensors, and the Nyquist plots obtained for increasing concentrations can be seen in Fig. 5a. The equivalent circuits employed for fitting the EIS data from Fig. 5a are detailed in Table S3, while the model-generated data for all the components integrated into the circuit can be found in Table S4. The modeling using the function available in the Nova software allowed for the uniform assessment of the R_ct_ value, which was subsequently used for calibration. It can be observed that the increase in concentration leads to an increase in R_ct_, demonstrating the successful immobilization of the cells on the electrode surface. The difference in R_ct_ after and before cell incubation (ΔR_ct_ = R_ct_ after cell incubation — R_ct_ after BSA blocking) was calculated and represented versus the concentration of cells to obtain the calibration curve (Fig. 5b). The variation is linear on the whole tested domain, and the calibration plot is described by the equation: ΔR_ct_ (Ω) = 0.01[HepG2 cells](cells/mL) + 161.99 and has a correlation coefficient of 0.985. Since the noise in EIS cannot be determined, it was considered that the limit of detection (LOD) is equal to the limit of quantification (LOQ), represented by the lowest concentration in the calibration domain, 10 cells/mL. Although the LOD does not cover the inferior domain of the concentration range in which circulating tumor cells can be found in blood (1–3000 cells/mL) [5], it covers a sufficiently large part of the domain, demonstrating its applicability as a proof-of-concept method for the screening of HCC. Moreover, the LOD is similar or even lower than other LODs reported in the literature, with only two studies reporting lower LODs for HepG2 cell detection using electrochemical aptasensors [5, 13].

The analytical performance of the developed aptasensor was compared to that of other electrochemical aptasensors from the literature (Table S5). Most examples found used the same aptamer, TLS11a, for electrode functionalization, but the detection was carried out using sandwich assays and voltammetric methods (differential pulse voltammetry). Nanoprobes functionalized with horseradish peroxidase, hemin, and G-quadruplex DNA sequences with enzyme-like activity were used for signal enhancement in these studies. In the case of this new approach, impedimetric detection requires no signal amplification strategy, making aptasensor fabrication simpler, faster, and cost effective. However, data analysis can be more cumbersome in the case of our method, since data fitting of Nyquist plots requires longer times. Moreover, aptamer immobilization was generally performed via sulfur-gold covalent bond formation by incubating the electrode with the aptamer solution for time intervals between 3 and 16 h. Our proposed method uses multipulse amperometry to facilitate the formation of amide bonds between the activated carboxyl groups and the amino groups in the structure of the aptamer, greatly reducing aptamer immobilization time to only 3 min. This allows faster and easier aptasensor production.

### Real sample analysis

For real sample analysis, undiluted commercial human serum samples were spiked with known concentrations of HepG2 cells. Suspensions of five different cell concentrations (10,000, 25,000, 50,000, 100,000, and 200,000 cells/mL) were prepared in human serum and incubated with the aptasensors. This protocol was used because due to the envisaged single-use application of the sensor, the standard addition method could not be performed. After incubation, EIS measurements were performed in 2-mM [Fe(CN)_6_]^4−/3−^, and the ΔR_ct_ values were calculated and plotted against the cell concentration. The resulting calibration curve is represented in Fig. 5c and is characterized by the equation: ΔR_ct_ (Ω) = 0.009[HepG2 cells](cells/mL) + 299.98 and a correlation coefficient of 0.975. It can be noticed that the sensitivity of the method applied for the detection of HepG2 from PBS (0.01 Ω × mL/ cells) and from serum (0.009 Ω × mL/ cells) is comparable, indicating a reduced matrix effect of only 5.26%. This demonstrates the applicability of the sensor for the analysis of complex matrices without the need for sample pre-treatment or dilution. Based on the calibration curve, undiluted serum samples were spiked with known concentration of cells, and the recoveries were calculated and presented in Table S6.

### Selectivity

The selectivity of the aptasensor for HepG2 cells was demonstrated by the immobilization of a random DNA aptamer on the platform, followed by blocking and incubation with HepG2 cells at 25,000 cells/mL and 100,000 cells/mL, respectively. The data obtained is represented in Fig. 5d. The incubation of HepG2 cells with the random sequence aptasensor led to no significant changes in the measured R_ct_ (7% increase in signal in the case of 25,000 cells/mL and –8.4% in the case of 100,000 cells/mL). Moreover, it can be noticed that the ΔR_ct_ is not proportional to the concentration of cells in this case, indicating that the slight changes observed are due to nonspecific adsorption on the electrode surface, not due to conformational changes of the aptamer.

The selectivity of the platform was further tested by incubating the aptasensors with fibroblasts (healthy cells used as negative control) and human lung adencocarcinoma A549 cells at a concentration of 200,000 cells/mL in PBS, followed by EIS analysis. The data obtained is represented in Fig. 5e. In this figure, the Nyquist plots obtained after the following steps are presented: BSA blocking with a 0.5% BSA solution (*light green line*), fibroblast immobilization for 30 min from a suspension of 200 000 fibroblasts/mL (*dark green line*), A549 immobilization for 30 min from a suspension of 200,000 cells/mL (*blue line*), and HepG2 cell immobilization for 30 min from a suspension of 200,000 HepG2 cells/mL (*orange line*). It can be seen from the figure that fibroblast and A549 cells immobilization leads to no significant changes in the Nyquist plot compared to the BSA blocking step (ΔR_ct_ = 177 Ω and 301, Ω, respectively, corresponding to a 2.03% and 3.45% increase). However, after HepG2 cell immobilization, a marked increase in R_ct_, can be observed (ΔR_ct_ = 1507 Ω). This behavior demonstrated the selectivity of the platform for HepG2 cells. Because the aptamer used is specific for these cells, its conformational change takes place only in the presence of its target, leading to changes in electrode surface resistance.

The affinity of the aptamer for HepG2 cells was assessed using isothermal titration calorimetry (ITC). It was observed that there was a strong interaction between the aptamer and the cells, which was not observed when testing the aptamer in the absence of cells. In addition, the thermal effect varies proportionally with the concentration (data not presented), thus proving the affinity between the two species.

### Stability

The stability of the aptasensors was tested by producing multiple aptasensors on the same day and testing them on consecutive days, on days 1, 2, 3, 6, and 7 (Fig. 5f). The aptasensors were kept in the fridge until testing. The aptasensors retained 97.8% of their initial signal on day 1, 96.2% on day 3, 96% on day 6, and 95.4% on day 7, demonstrating good stability for up to a week. The repeatability of a single sensor was not tested, since the application of the aptasensor is envisaged for single use.

## Conclusions

A novel impedimetric aptasensor based on the TLS11a aptamer was developed for the detection of HepG2 HCC tumor cells. The aptasensing platform was based on carbon screen-printed electrodes functionalized with graphene oxide, chitosan, and nanopolymeric polyaniline particles. The carboxylic groups in the structure of GO were activated to allow the immobilization of the amino-terminated TLS11a aptamer, followed by blocking and HepG2 cell incubation. The immobilization of the aptamer was performed using a multipulse-assisted procedure, which was applied here for the first time for the immobilization of aptamers via amide bond formation. The successful aptamer and cell immobilization were confirmed by SEM, AFM, and XPS analyses, as well as by EIS and CV. HepG2 cells were detected using a label-free impedimetric method, based on calculating the difference in R_ct_ after and before cell incubation and plotting it against the cell concentration to obtain the calibration curve. The aptasensor demonstrated great selectivity and stability and proved suitable for the analysis of undiluted serum samples containing HepG2 cells, paving the way for the development of new early diagnosis tools for hepatocellular carcinoma. For clinical applications, it has to be taken into account that some circulating tumor cells in patients’ blood can have a different polymorphism compared to HepG2 cells. In this case, the TLS11a aptamer in the aptasensor design can be easily changed with another aptamer that is selective for the respective cells. The advantages of the presented method are the innovative multipulse method for aptamer immobilization, which greatly reduces aptasensor fabrication times, as well as the signal amplification-free detection, which renders the method more cost and time efficient.

Author contribution

AP, conceptualization, data curation, formal analysis, investigation, methodology, writing — original draft, and writing — review and editing. MT, conceptualization, data curation, formal analysis, investigation, methodology, writing — original draft, writing — review and editing, and resources. DK, formal analysis, investigation, and methodology. DB, data curation, formal analysis, and methodology. MS, data curation, formal analysis, and methodology. OP, data curation, formal analysis, and methodology. IF, data curation, investigation, and methodology. FG, conceptualization and writing — review and editing. CC, conceptualization, project administration, resources, supervision, and writing — review and editing. NAH, conceptualization, supervision, and writing — review and editing.

### Electronic supplementary material

Below is the link to the electronic supplementary material.

Supplementary Material 1
