# Supplementary material for: Label-free electrochemical aptasensor for the detection of HepG2 hepatocellular carcinoma cells
Source: Mikrochim Acta. 2024 Jun 21;191(7):413. doi: 10.1007/s00604-024-06479-x (PMC11192815; doi:10.1007/s00604-024-06479-x)
Supplement: Supplementary file 1 — Supplementary Material 1 [file 604_2024_6479_MOESM1_ESM.docx]

**Supplementary Information**

**Label-free electrochemical aptasensor for the detection of HepG2 hepatocellular carcinoma cells**

Alexandra Pusta^1,2^, Mihaela Tertiș^1^*, Denisa Kezan^1^, Diana Bogdan^3^, Maria Suciu^3^, Ovidiu Pană^3^, Ionel Fizeșan^4^, Florin Graur^5^, Cecilia Cristea^1^*, Nadim Al-Hajjar^5^

^1^Iuliu Hațieganu” University of Medicine and Pharmacy, Department of Analytical Chemistry, 4 Pasteur Street, 400349, Cluj-Napoca, Romania [alexandra.pusta@umfcluj.ro](mailto:alexandra.pusta@umfcluj.ro), [mihaela.tertis@umfcluj.ro](mailto:mihaela.tertis@umfcluj.ro), [denisa.miha.kezan@elearn.umfcluj.ro](mailto:denisa.miha.kezan@elearn.umfcluj.ro), [ccristea@umfcluj.ro](mailto:ccristea@umfcluj.ro)

^2^Iuliu Hațieganu” University of Medicine and Pharmacy, Department of Medical Devices, 4 Pasteur Street, 400349, Cluj-Napoca, Romania

^3^National Institute for Research and Development of Isotopic and Molecular Technologies, 67-103 Donath Street, 400293, Cluj-Napoca, Romania [diana.bogdan@itim-cj.ro](mailto:diana.bogdan@itim-cj.ro), [suciu.maria@ubbcluj.ro](mailto:suciu.maria@ubbcluj.ro), [ovidiu.pana@itim-cj.ro](mailto:ovidiu.pana@itim-cj.ro)

^4^Electron Microscopy Centre “C. Craciun”, Biology and Geology Faculty, Babes-Bolyai University Cluj-Napoca, 5-7 Clinicilor Str. 400006 Cluj-Napoca, Romania,

^5^Iuliu Hațieganu” University of Medicine and Pharmacy, Department of Toxicology, 8 Victor Babeș, 400012, Cluj-Napoca, Romania [Ionel.Fizesan@umfcluj.ro](mailto:Ionel.Fizesan@umfcluj.ro)

^6^Iuliu Hațieganu” University of Medicine and Pharmacy, Department of Surgery 3, 19-21 Croitorilor, 400162, Cluj-Napoca, Romania [florin.graur@umfcluj.ro](mailto:florin.graur@umfcluj.ro), [na_hajjar@yahoo.com](mailto:na_hajjar@yahoo.com)

[*ccristea@umfcluj.ro](mailto:*ccristea@umfcluj.ro) and [mihaela.tertis@umfcluj.ro](mailto:mihaela.tertis@umfcluj.ro)

**Materials and methods**

1. **Materials**
   1. **Chemicals and reagents**

Aptamer TLS11a with the following sequence 5'-ACA-GCA-TCC-CCA-TGT-GAA-CAA-TCG-CAT-TGT-GAT-TGT-TAC-GGT-TTC-CGC-CTC-ATG-GAC-GTG-CTG-3’ (K_d_ = 4.51 ± 0.39 nM) (Shangguan et al., 2008) RP-HPLC purity was purchased from [Eurogentec](https://www.eurogentec.com/en/) (Seraing, Belgium). The 5’ end of the aptamer was functionalized with an amino group and a sequence of six carbon atoms between the amino group and the DNA sequence, thus ensuring that a sufficient distance is maintained between the aptamer and the electrode surface. Graphene oxide (GO) suspension 4 mg/mL in water was purchased from [Metrohm DropSens](https://www.dropsens.com/), Spain. Bovine serum albumin (BSA) was purchased from [Glentham Life Sciences, UK.](https://www.glentham.com/en/)

Cancerous hepatic cells (HepG2),normal human foreskin fibroblasts (BJ cells) and human lung adenocarcinoma A549 cells purchased from [ATCC](https://www.atcc.org/about-us?matchtype=&network=x&device=c&adposition=&keyword=&gad_source=1&gclid=CjwKCAiAuNGuBhAkEiwAGId4atDRczgR4lfOhPtVrHUPJgmaGshX1jqFes0jIneJzpleNvxz7PlEkBoCAV0QAvD_BwE) (Manassas, United States of America) were used to evaluate the performance of the aptasensor and its specificity towards tumor cells. Eagle's Minimum Essential Medium (EMEM) with low glucose (1 g/L), Dulbecco's Modified Eagle Medium (DMEM) with low glucose (1 g/L) and high glucose (4.5 g/L), fetal bovine serum (FBS), and phosphate buffer saline (PBS) ([Gibco](https://www.thermofisher.com/ro/en/home/life-science/bioproduction/gibco-bioprocessing.html?gclid=CjwKCAiAuNGuBhAkEiwAGId4ahg9W9fIv6C7p4hjYbN4YEeSqcty5_BMWofi-rbyclgXnCUcB5xV3hoCkTUQAvD_BwE&cid=bpd_cct_ccs_r01_co_cp1494_pjt9122_bpd00000_0se_gaw_ta_awa_Universal&ef_id=CjwKCAiAuNGuBhAkEiwAGId4ahg9W9fIv6C7p4hjYbN4YEeSqcty5_BMWofi-rbyclgXnCUcB5xV3hoCkTUQAvD_BwE:G:s&s_kwcid=AL!3652!3!654633970476!b!!g!!gibco%20cell%20culture%20media!12582389685!118427355094&gad_source=1), NY, USA) were used for cell culture and experimentation.

Commercial human serum was obtained form [Sigma-Aldrich](https://www.sigmaaldrich.com/RO/en). DNAse and RNAase free water were obtained from Invitrogen, USA. All other reagents were purchased [from Sigma-Aldrich](https://www.sigmaaldrich.com/RO/en), [Fluka Chemie](https://www.analytics-shop.com/gb/fluka) GmbH, and [Merck Chemicals](https://www.merck.com/). All reagents were of analytical grade and were used as received, without further purification. All solutions were prepared in DNAase and RNAase free water ([Invitrogen](https://www.thermofisher.com/ro/en/home/brands/invitrogen.html), USA), unless mentioned otherwise.

- 1. **Instruments**

A multichannel potentiostat/galvanostat Autolab MAC80100 ([Metrohm](https://www.metrohm.com/ro_ro.html), Utrecht, The Netherlands) operated with Nova 1.10.4 software was used to perform all the electrochemical tests. The electrodes used were carbon screen-printed electrodes (C-SPE) with a silver pseudo-reference and a carbon auxiliary electrode ([Metrohm DropSens](https://www.dropsens.com/), Madrid, Spain).

Cell pelleting was done using a Sigma 2-16centrifuge (Osterode am Harz, Germany) while cell counting was done under an inverted optical microscope ([Zeiss](https://www.zeiss.com/corporate/en/home.html), Axiovert 40 CFL, Oberkochen, Germany) using counting Burker-Turk chambers.

Scanning electron microscopy (SEM) images were taken using a Hitachi SU8230 at 30 kV, 10 mA and 8 mm working distance.

Atomic force microscopy (AFM) images were obtained on a Cypher S ([Asylum Research](https://afm.oxinst.com/), Santa Barbara, CA) microscope in the air.

The qualitative sample compositions were investigated using X-Ray Photoelectron Spectroscopy (XPS) assisted by Ar ions etching. The XPS spectra were recorded using a SPECS spectrometer working with an Mg anode (1253.64 eV) as X-rays source. The spectra were analyzed by using CASA XPS software.

Isothermal titration calorimetry (ITC) was performed using an Affinity ITC calorimeter ([TA Instruments](https://www.tainstruments.com/products/microcalorimetry/?gad_source=1&gclid=CjwKCAiAuNGuBhAkEiwAGId4avyVIiAxApeLN6fcwUVmgnUod9fj8Pwip7A30x26hX_Pl93pPTA2QBoCrX8QAvD_BwE), Waters®, New Castle, USA). The data was analyzed using the NanoAnalyze software.

OriginPro 8.5 was used for graph representation and Biorender.com was used to create the graphical abstract.

1. **Methods**
   1. **Aptamer solution preparation**

The aptamer solution was prepared by dissolving the aptamer in the appropriate volume of 0.01 M TRIS pH 7.2 buffer (containing 0.1 M NaCl, 0.1 M KCl, and 0.01 M MgCl_2_) to obtain a 100 μM aptamer stock solution. The stock solution was aliquoted, and the aliquots were kept in the freezer until use. The aliquots were diluted to the desired concentration using TRIS buffer and a thermal denaturation protocol was applied before aptamer immobilization on the electrode as follows: the solution was kept at 95 ℃ for 5 minutes in a Thermomixer (Eppendorf, Germany), followed by a rapid cooling at – 20 ℃ for 30 seconds in the freezer. The aptamer dilutions were prepared daily before use.

- 1. **SEM, AFM and XPS characterization**

Samples were prepared for SEM imaging by sputter coating with a 10 nm layer of Pt/Pd (Agar Auto Sputer Coater). Images were taken from at least 3 different places on the sample at different magnifications. Before SEM analysis, the aptasensors incubated with HepG2 cells were fixed by incubating them with a 2.5% glutaraldehyde solution prepared in 0.01 M PBS pH 7.4 for 90 minutes at 4 ℃.

AFM measurements were performed in AC mode (tapping mode) using AC160TS-R3 silicon cantilevers (Olympus) with a spring constant of ~26 N/m and a resonance frequency of ~300 kHz. Multiple areas of the samples were analyzed at different scan sizes, with 512 pixels/line and with a scan rate of less than 1 Hz. Image analysis was performed using the integrated Asylum Research software (AR16, Asylum Research) written within Igor Pro software package (Igor Pro 6, WaveMetrics, Inc., Lake Oswego, OR, USA).

In the case of XPS, to remove the molecular species resulting from contamination, we used a cleaning sputtering with accelerated Ar ions at 500 V for 5 minutes. Pieces of 5x5 mm were cut from each sample electrode and mounted on the sample holder by using carbon tape. The integral intensities were calculated by dividing the recorded data at relative intensities, transmission factors and electron mean free paths from the CASA database. A Shirley background was extracted in all the core-level spectra.

- 1. **Isothermal titration calorimetry**

Titrations were carried out using a 300 μL autopipette that was filled with 100 μM aptamer solution for the blank titration. The sample cell was filled with different concentrations of cell suspensions (1000 cells/mL and 100000 cells/mL), cell lysate, or phosphate buffer for the blank titration. The stirring speed was 100 rpm. The volume of injection was 2 μL for the first injection and 5 μL for the following 14 injections.

**Table S1.** The equivalent circuit models employed to fit the experimental data obtained from the protocol used in the development of the aptasensor (the data presented in Figure 1 B and Figure 1 C).

| **Aptasensor development step** | **Equivalent circuit - image** | **Equivalent circuit - notation** |
| --- | --- | --- |
| Bare C-SPE | **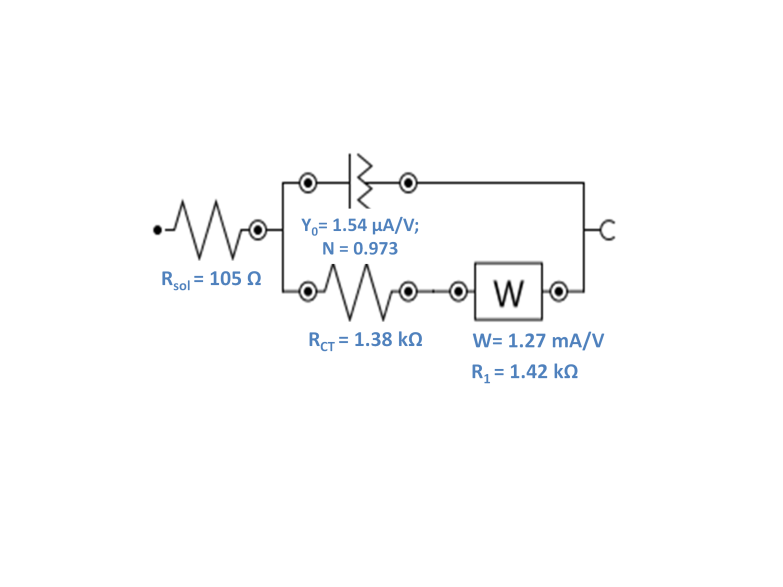** | [R_Sol_(Q[R_CT_W])] |
| C-SPE_GO/Chi/NP | **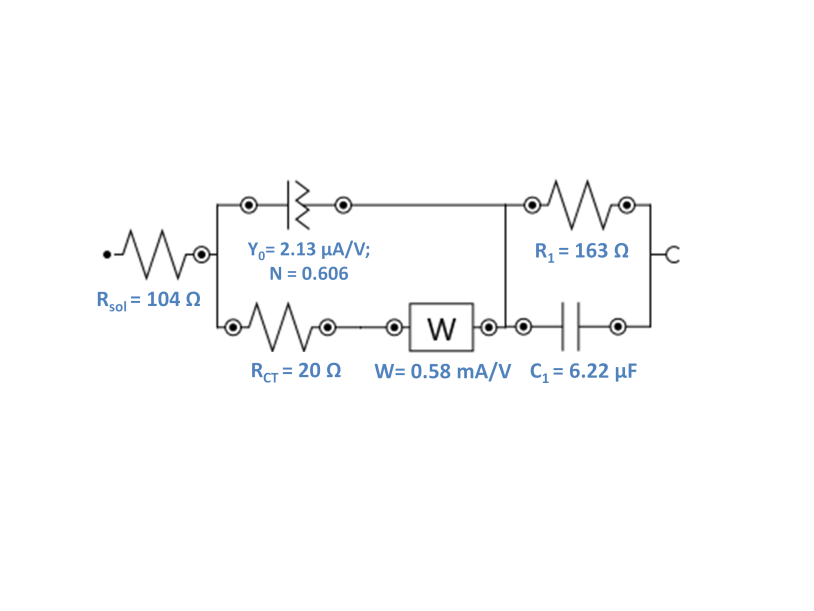** | [R_Sol_(Q[R_CT_W])(R_1_C_1_)] |
| Activated  C-SPE_GO/Chi/NP | **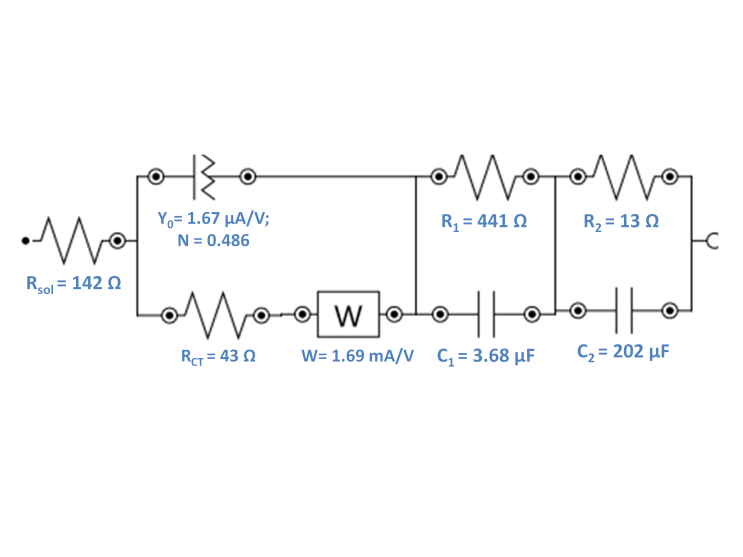** | [R_Sol_(Q[R_CT_W])(R_1_C_1_)(R_2_C_2_)] |
| C-SPE_GO/Chi/NP_Apt | **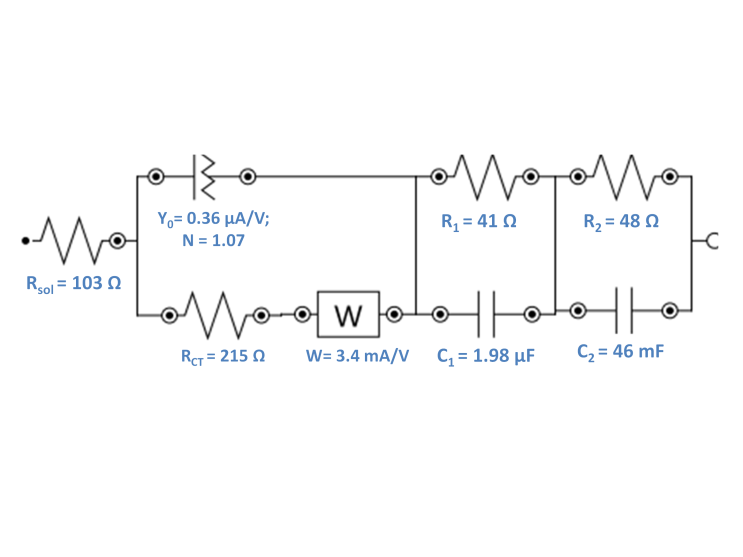** | [R_Sol_(Q[R_CT_W])(R_1_C_1_)(R_2_C_2_)] |
| C-SPE_GO/Chi/NP_Apt_BSA | **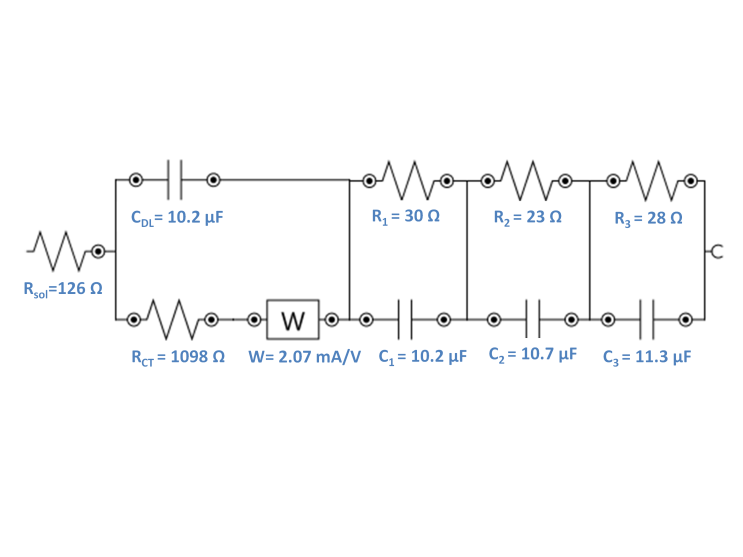** | [R_Sol_(C_DL_[R_CT_W])(R_1_C_1_)(R_2_C_2_)(R_3_C_3_)] |
| C-SPE_GO/Chi/NP_Apt_ BSA_HepG2 cells | **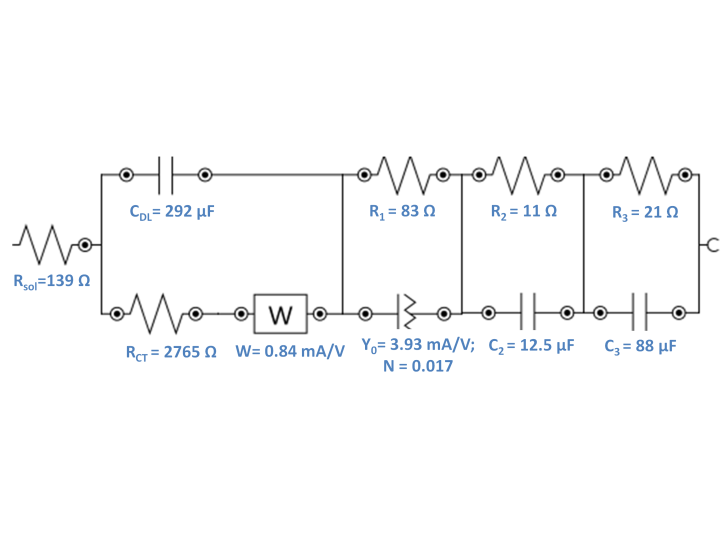** | [R_Sol_(C_DL_[R_CT_W])(R_1_Q_1_)(R_2_C_2_)(R_3_C_3_)] |

**Table S2.** The equivalent circuits used for fitting the experimental data from **Figure 1 B and Figure 1 C**

| **Aptasensor elaboration steps** | **R_S_ (Ω)** | **R_CT_ (kΩ)** | **CPE (µA/V)**  **N** | **C_DL_ (µF)** | **W (mA/V)** | **R_1_ (Ω)** | **C_1_ (µF)/Q1(mA/V)N** | **R_2_ (Ω)** | **C_2_ (µF)/Q2** | **R_3_ (Ω)** | **C_3_ (µF)** | **Χ^2^** |
| --- | --- | --- | --- | --- | --- | --- | --- | --- | --- | --- | --- | --- |
| Bare C-SPE | 105 | 1.380 | 1.54  0.973 | - | 1.27 | - | - | - | - | - | - | 0.0062 |
| C-SPE_GO/Chi/NPs | 104 | 0.020 | 2.13  0.606 | - | 0.58 | 163 | 6.22 | - | - | - | - | 0.0025 |
| Activated C-SPE_GO/Chi/NPs | 142 | 0.043 | 1.67  0.486 | - | 1.69 | 441 | 3.68 | 13 | 202 | - | - | 0.0119 |
| C-SPE_GO/Chi/NPs_Apt | 103 | 0.215 | 0.36  1.07 | - | 3.40 | 41 | 1.98 | 48 | 46000 | - | - | 0.0007 |
| C-SPE_GO/Chi/NPs_Apt_BSA | 126 | 1.098 | - | 10.2 | 2.07 | 30 | 10.2 | 23 | 10.7 | 28 | 11.3 | 0.0024 |
| C-SPE_GO/Chi/NPs_Apt_ BSA_HepG2 cells | 139 | 2.765 | - | 292 | 0.84 | 83 | 3.93  0.017 | 11 | 12.5 | 21 |  | 0.0096 |


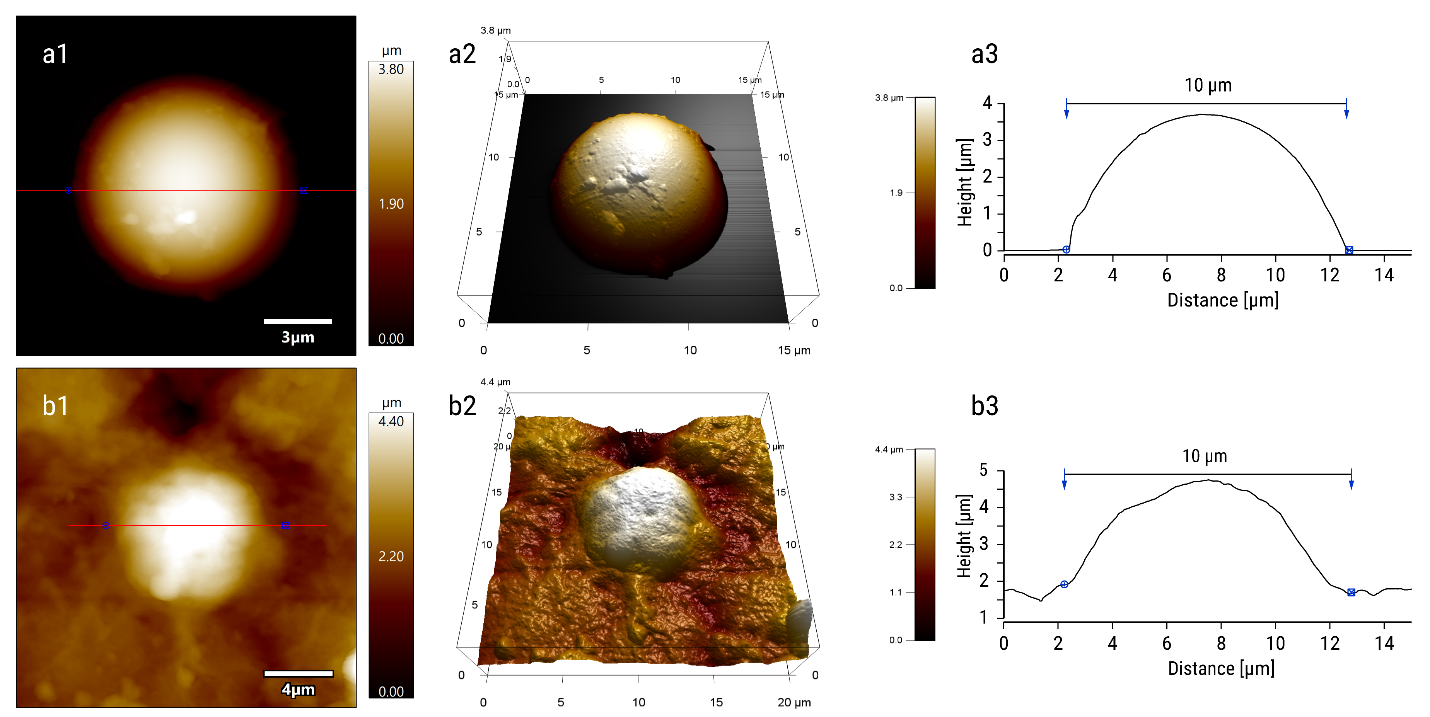


**Fig. S1** 2D (A1 and B1), 3D (A2 and B2) AFM images of C-SPE_GO/Chi/NPs_Apt with immobilized HepG2 cells and section profiles along the red line highlighted in the 2D images. The section profiles indicate a diameter of about 10 µm of the immobilized cells (two different cells are presented). Scan size 15 µm (A3; top) and 20 µm (B3; bottom)

**Table S3.** The equivalent circuits used for fitting the experimental data from **Figure 5 A.**

| HepG2 cells concentration  (cells mL^-1^) | **Equivalent circuit** |
| --- | --- |
| 0 | **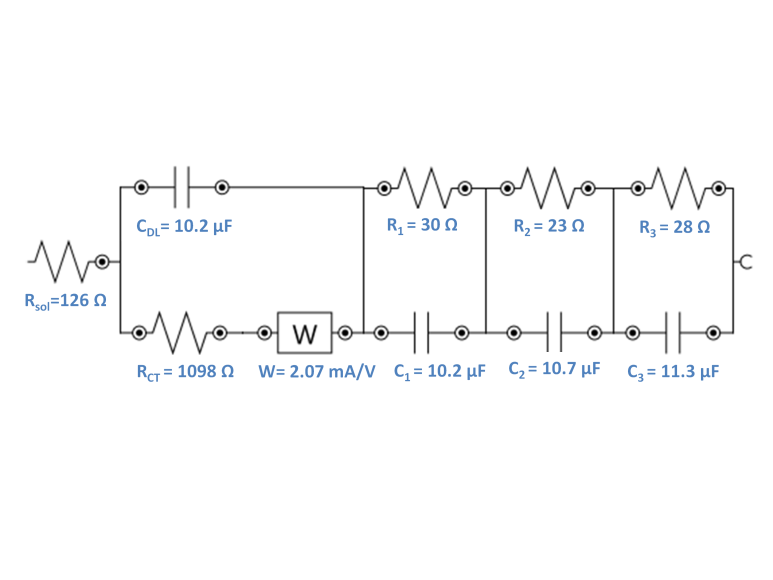** |
| 10 | 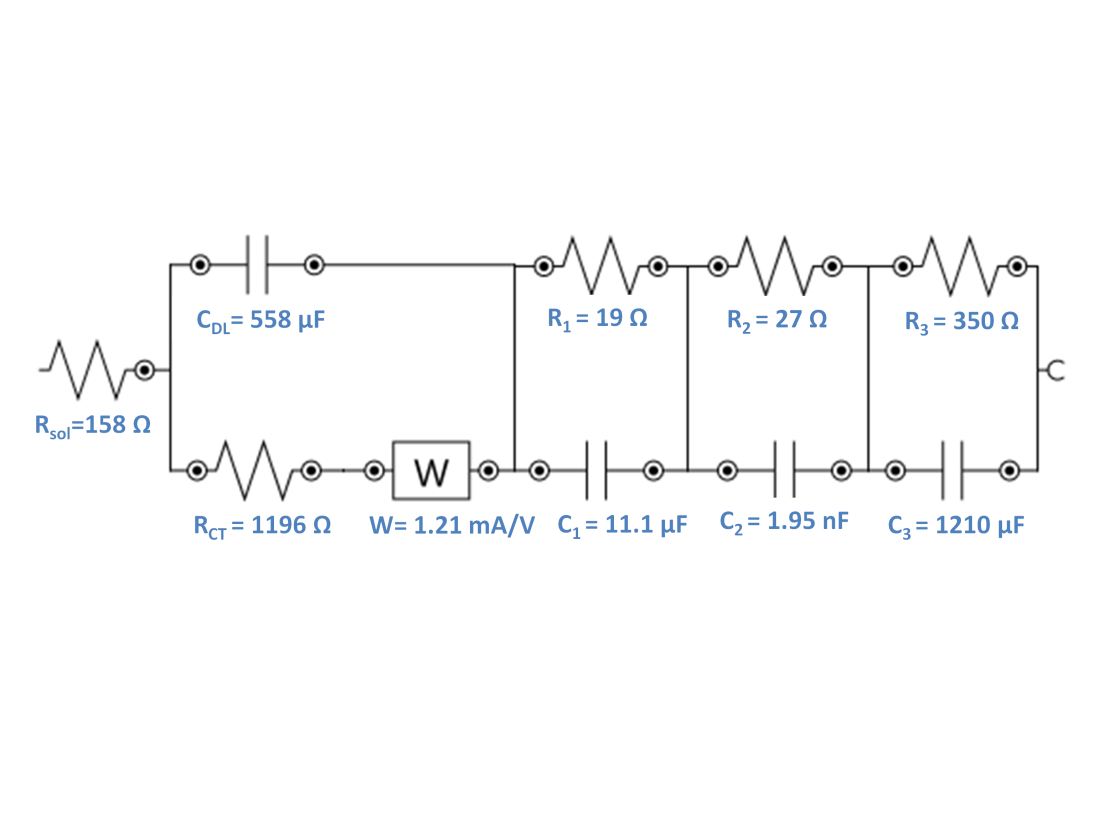 |
| 1000 | 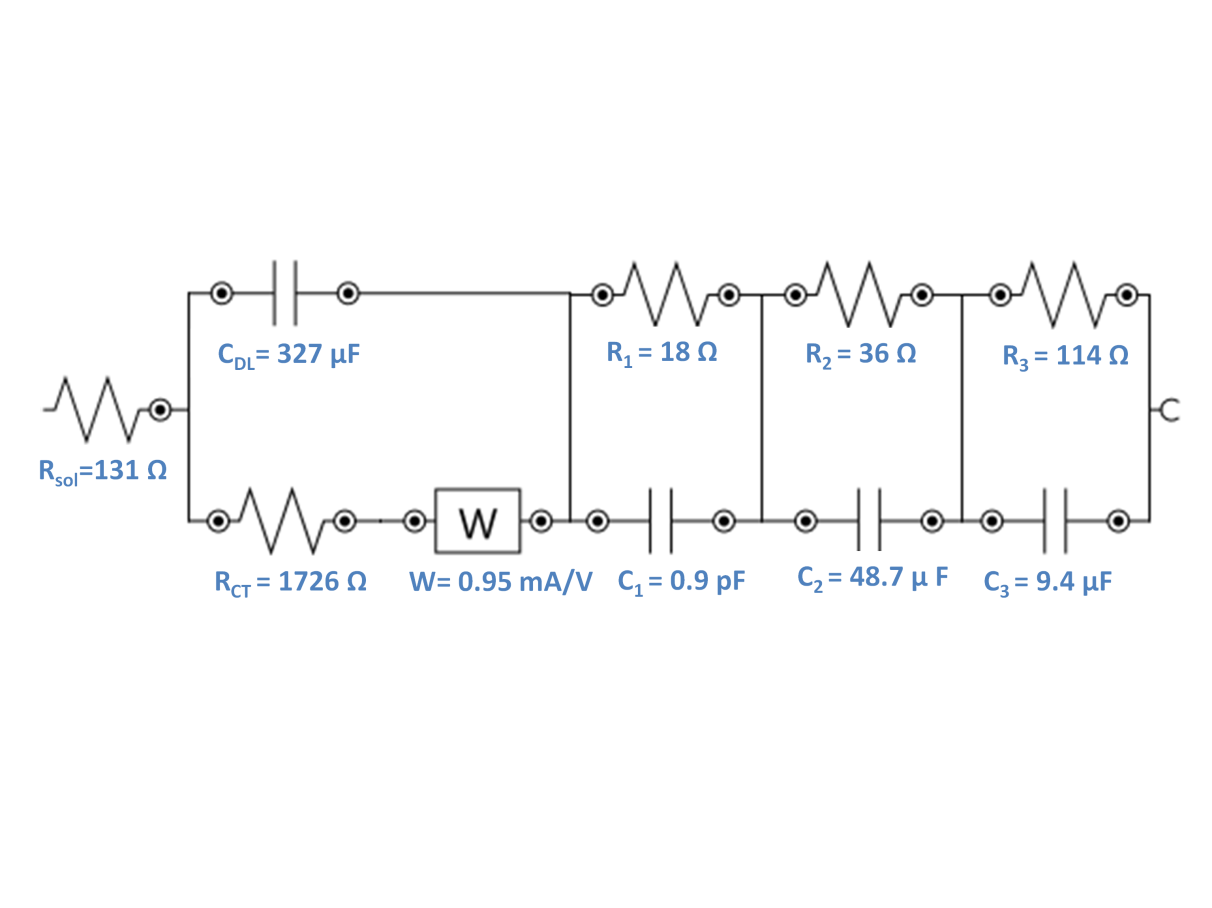 |
| 10000 | 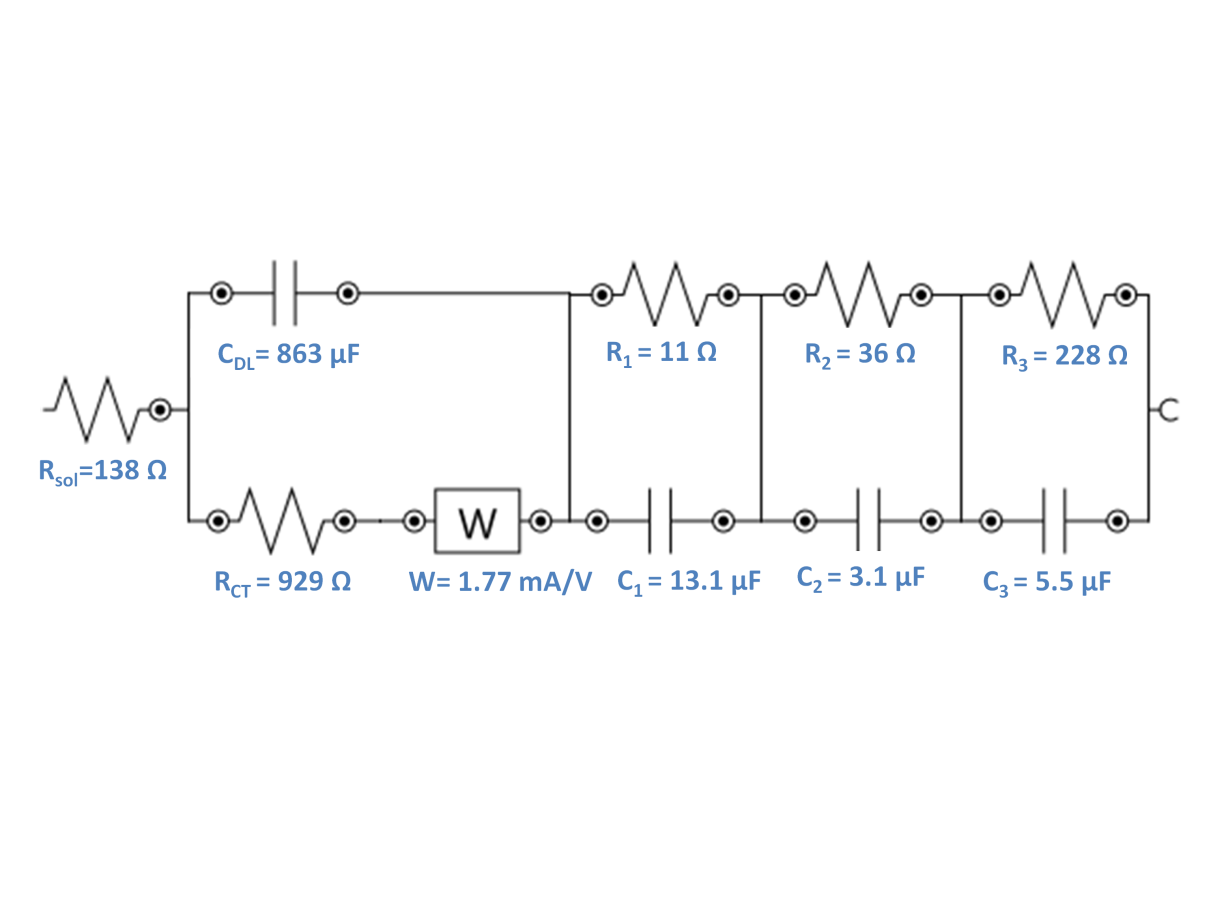 |
| 25000 | 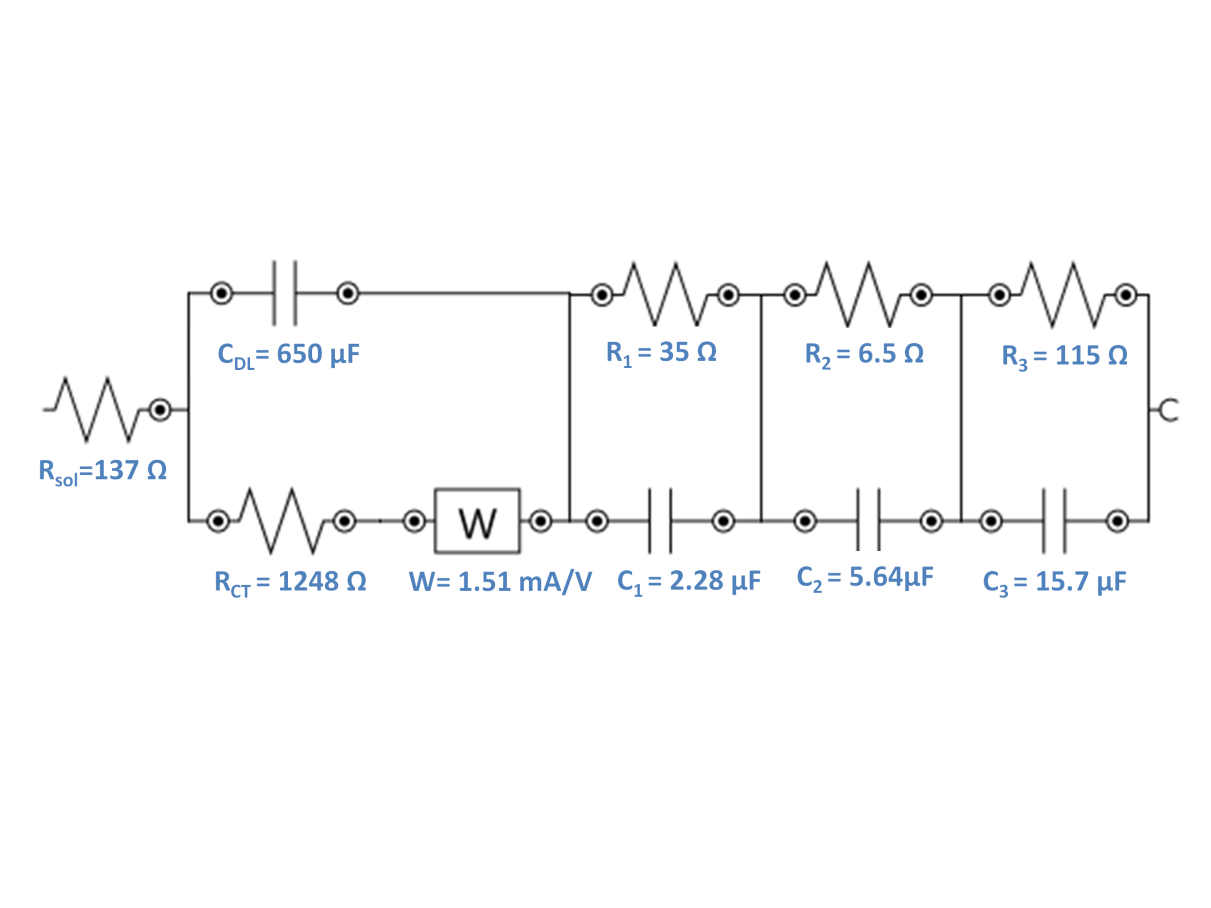 |
| 50000 | 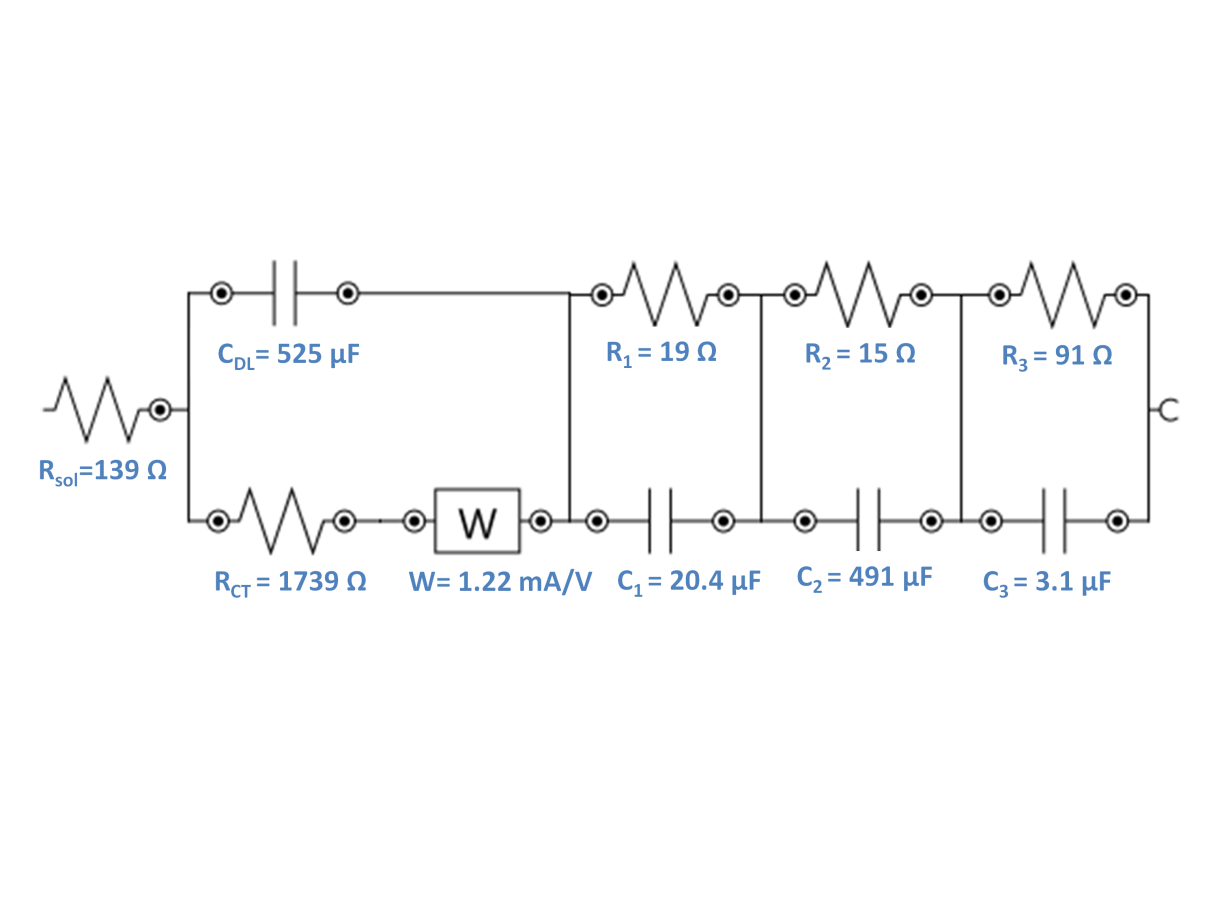 |
| 100000 | 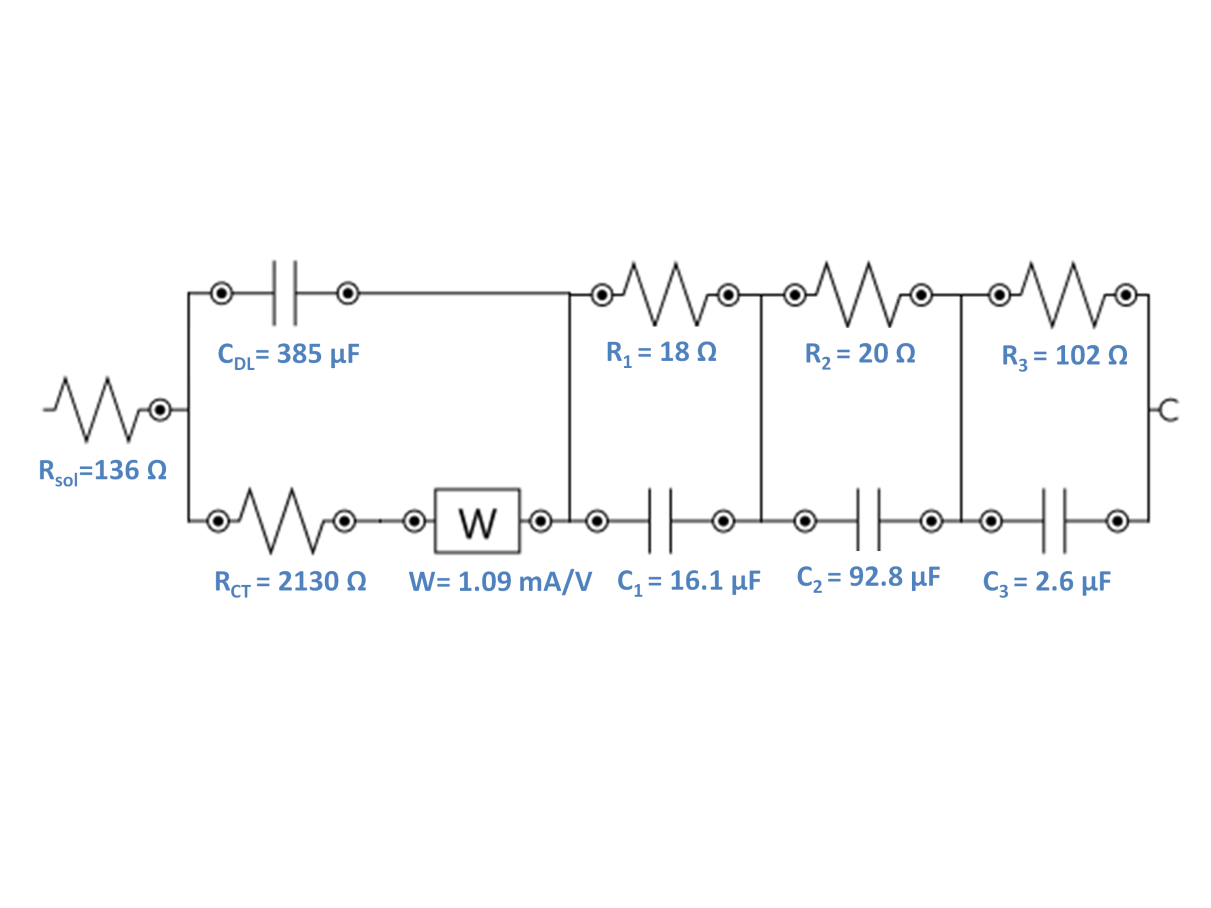 |
| 200000 | 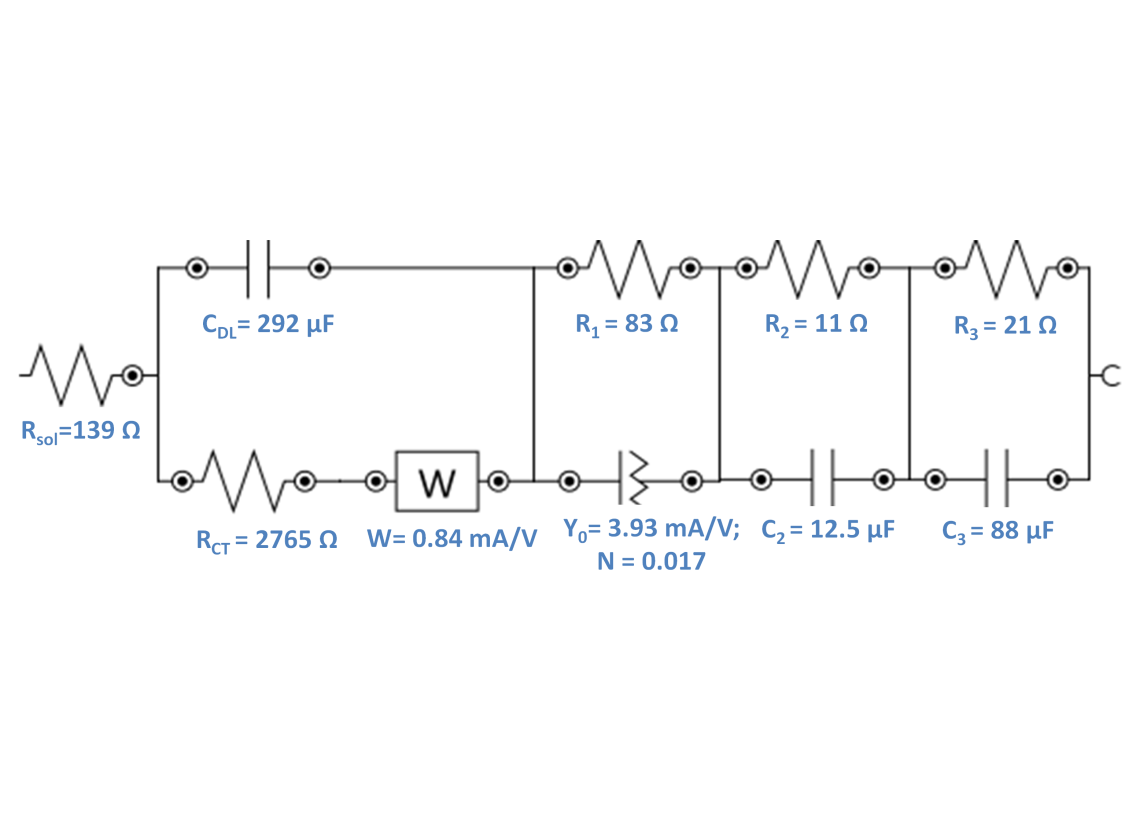 |

**Table S4.** Values of the EIS parameters from the equivalent circuit obtained with the fitting and simulation option of the software applied for **Figure 5A**.

| **HepG2 cells conc.**  **(cells mL-1)** | **R_S_ (Ω)** | **R_CT_ (Ω)** | **C_DL_ (µF)** | **W (mA/V)** | **R_1_ (Ω)** | **C_1_ (µF)/**  **Q1(mA/V)**  **N** | **R_2_ (Ω)** | **C_2_ (µF)** | **R_3_ (Ω)** | **C_3_ (µF)** | **χ^2^** |
| --- | --- | --- | --- | --- | --- | --- | --- | --- | --- | --- | --- |
| 0 | 126 | 1.098 | 10.2 | 2.07 | 30 | 10.2 | 23 | 10.7 | 28 | 11.3 | 0.0024 |
| 10 | 158 | 1.196 | 558 | 1.21 | 19 | 11.1 | 31 | 0.002 | 350 | 1210 | 0.0140 |
| 1000 | 131 | 1.726 | 327 | 0.95 | 18 | 0.9 | 36 | 48.7 | 114 | 9.4 | 0.0079 |
| 10000 | 138 | 0.93 | 863 | 1.77 | 11 | 13.1 | 36 | 3.1 | 228 | 5.5 | 0.0042 |
| 25000 | 137 | 1.248 | 650 | 1.51 | 35 | 2.28 | 6.5 | 5.64 | 115 | 15.7 | 0.0017 |
| 50000 | 139 | 1.739 | 525 | 1.22 | 19 | 20.4 | 15 | 491 | 91 | 3.1 | 0.0018 |
| 100000 | 136 | 2.130 | 385 | 1.09 | 18 | 16.1 | 20 | 92.8 | 102 | 2.6 | 0.0017 |
| 200000 | 139 | 2.765 | 292 | 0.84 | 83 | 3.93  0.017 | 11 | 12.5 | 21 | 88 | 0.0096 |

**Table S5**. Comparison of the analytical performance of the developed aptasensor with aptasensors published in the literature

| **Aptasensor** | **Amplification strategy** | **Detection** | **Aptamer** | **Linear range (cells/mL)** | **LOD**  **(cells/mL)** | **Aptamer incubation time** | **Reference** |
| --- | --- | --- | --- | --- | --- | --- | --- |
| AuE/Apt + nnoprobe | Enzymatic signal amplification  Sandwich assay with nanoprobe: G-quadruplex/hemin/Apt–AuNPs–HRP) | Voltammetric | TLS11a | 1x10^2^ – 1x10^7^ | 30 | **10 h** | (Sun et al., 2015b) |
| ITO/AuNP/Apt | Enzymatic signal amplification  Sandwich assay with nanoprobe: G-quadruplex/hemin/Apt– ZnO@Au-Pd –HRP) | Voltammetric | TLS11a | 1x10^2^ – 1x10^7^ | 10 | **16 h** | (Sun et al., 2017) |
| AuE/DNA nanotetrahedron | Multibranched hybridization chain  reaction  G-quadruplex/hemin/Apt– MIL-101@AuNPs–HRP) | Voltammetric | TLS11a | 1x10^2^ – 1x10^7^ | 5 | **Overnight** | (Chen et al., 2018) |
| AuE/DNA nanotetrahedron | Enzymatic signal amplification  Sandwich assay with nanoprobe: G-quadruplex/hemin/Apt–Pt-Pd –HRP) | Voltammetric | TLS11a | 10 – 1x10^6^ | 5 | **2 h** | (Sun et al., 2018) |
| **C-SPE/GO/Chi/NP_Apt** | **No amplification** | **Impedimetric** | **TLS11a** | **10 – 2x10^6^** | **10** | **3 min** | **This work** |

AuE – gold electrode; Apt – aptamer; AuNP – gold nanoparticle; HRP – horseradish peroxidase; ITO – indium tin oxide electrode; ZnO@Au-Pd - Zn O gold/palladium-functionalized nanorods; GO – graphene oxide; Chi – chitosan;

**Table S6**. Recoveries obtained for the analysis of spiked human serum samples

| **Spiked cell concentration (cells/mL)** | **ΔR_ct_** | **Recovery (%)** |
| --- | --- | --- |
| 10000 | 306 | 115.04 |
| 25000 | 542 | 95.20 |
| 50000 | 685 | 95.74 |
| 100000 | 1335 | 105.03 |
| 200000 | 1948 | 93.52 |
